# Comparative transcriptomics of two petal variants reveals key functional genes underlying petal shape development in lotus (*Nelumbo*)

**DOI:** 10.3389/fpls.2025.1596925

**Published:** 2025-07-08

**Authors:** Jiaxin He, Yini Ma, Qingqing Liu, Rui Zhang, Guohong Huang, Dasheng Zhang, Fengluan Liu, Caixia Yang

**Affiliations:** ^1^ Liaoning Key Laboratory of Urban Integrated Pest Management and Ecological Security, College of Life Science and Bioengineering, Shenyang University, Shenyang, China; ^2^ Shanghai Key Laboratory of Plant Functional Genomics and Resources, Shanghai Chenshan Botanical Garden, Shanghai, China

**Keywords:** epidermal cell, lotus, *Nelumbo*, *N. lutea*, *N. nucifera*, petal shape, transcriptome

## Abstract

**Introduction:**

The lotus (*Nelumbo* Adans.) is a versatile plant that integrates ornamental beauty, culinary applications, medicinal benefits, ecological significance, and cultural symbolism. However, its ornamental value is somewhat restricted by the relatively limited diversity in petal shapes. Consequently, it is essential to explore the genes regulating petal shape, in order to lay a primary foundation for molecular-assisted breeding of lotus cultivars with novel petal shapes.

**Methods:**

This study focused on two variants with distinct petal shapes: the broad petals of *N. lutea* M512 and the narrow petals of *N. nucifera* ‘Chenshan Feiyan’ (CSFY). Petal shape differences, including length, width, length-to-width ratio, and epidermal cell density, were compared at four floral bud stages between these variants and their respective wild types. Using RNA-sequencing technology, differentially expressed genes (DEGs) between variant and wild-type petals were identified, followed by gene ontology (GO) and kyoto encyclopedia of genes and genomes (KEGG) enrichment analyses. By integrating the results of morphological and enrichment analysis, key genes involved in the development of wide and narrow petal shapes in lotus were identified.

**Results:**

It revealed that the broad petal variation of M512 was caused by a reduction in petal length while maintaining width, whereas the narrow petal phenotype of CSFY resulted from a combination of increased length and decreased width. The final petal shapes in both variants were primarily determined by the total number of cells along the petal’s longitudinal (length) and transverse (width) directions, rather than by cell size or shape. A total of 59 and 96 candidate genes associated with petal shape development were identified in broad-petaled M512 and narrow-petaled CSFY, respectively. Many of these genes are directly involved in the development of cell wall/membrane and in the synthesis and metabolic pathways of plant hormones such as cytokinins, auxins, jasmonic acid, and brassinosteroids.

**Discussion:**

The main framework for petal shape was established during stages D1 and D5. The key genes identified in this study will facilitate the development of artificial techniques for petal shape regulation and serve as a theoretical foundation for molecular-assisted breeding in lotus.

## 1 Introduction

Lotus (*Nelumbo* Adans.) is one of the ten traditional famous flowers in China, and it is also the national flower of India and Vietnam and a famous religious flower worldwide. The lotus rhizome is an important vegetable in China, while lotus seeds, flowers, and leaves have medicinal applications. Additionally, lotus also plays a role in the purification of aquatic ecosystems. Thus, the lotus is a multifunctional plant integrating ornamental, edible, medicinal, ecological, and cultural values (Wang and Zhang, 2004; Chen et al., 2019; Lin et al., 2019). To date, the genus *Nelumbo* comprises only 2 species: the Asian lotus (*N. nucifera* Gaertn.), which is widely distributed in Asia and northern Australia, with nearly 2,000 known cultivars; and the American yellow lotus (*N. lutea*), native to North and Central America, which remains largely in a semi-wild or naturalized state and is characterized by single-petaled, pale yellow flowers (Li et al., 2014; Liu et al., 2023).

Petal shape is a key component of floral morphology and has a significant impact on the ornamental value of flowering plants. Although 95.6% of the 2080 documented lotus cultivars are ornamental types (Liu et al., 2019), the petal shape of modern lotus shows limited diversity due to the monotony of ancestral petal morphology, the lack of targeted breeding efforts, and the absence of breakthroughs in molecular assisted breeding techniques. Most lotus petal shapes are intermediate forms, such as oval, spoon-shaped, or broad lanceolate forms, lacking extreme traits such as the broad petals of roses or the ray petals of chrysanthemums, which reduces their market competitiveness. Over the past decade, our team has collected and preserved over 700 lotus germplasm resources, leading to the identification of 2 stable petal shape variants: M512, a broad-petal variant derived from a natural seedling mutation of the American lotus (Liu et al., 2020), and ‘Chenshan Feiyan’ (CSFY), a narrow-petal cultivar created through ^60^Coγ radiation mutagenesis of the seeds of the Asian lotus Weishan Hong (Liu et al., 2021). Both variants provide valuable materials for elucidating the mechanisms of petal shape formation and developing novel lotus cultivars with extreme petal shapes.

In the research on the development of plant petal morphology, few studies have specifically addressed variations in petal shape such as broadness or narrowness, with most focusing on the regulation of petal size or growth during natural development. Early studies primarily used *Arabidopsis* as a model, identifying several regulatory genes involved in petal size and growth, including *AINTEGUMENTA* (Mizukami and Fischer, 2000), *AUXIN RESPONSE FACTOR* 2 (Schruff et al., 2006), *BIG BROTHER* (Disch et al., 2006), *BIGPETAL* (Szécsi et al., 2006), *P450 KLUH*/*CYP78A5* (Anastasiou et al., 2007), *AUXIN RESPONSE FACTOR8* (Varaud et al., 2011), *JAGGED* (Sauret-Güeto et al., 2013), and *SPIKE1* (Ren et al., 2016). Recently, genes regulating petal size and petal elongation have also been identified in major ornamental crops such as *Gerbera hybrida* (Han et al., 2017; Huang et al., 2020), chrysanthemums (Guan et al., 2022), and roses (Jing et al., 2023; Wang et al., 2024; Jin et al., 2025), providing theoretical support for the development of new cultivars with desirable petal shapes. Among these genes, a few have shown effects on petal shape. For instance, the *Arabidopsis* mutant of *AINTEGUMENTA* exhibited narrow petals (Elliott et al., 1996), while the *SPIKE1* knockout mutant developed longer and thinner petals (Ren et al., 2016). Similarly, mutations in *RABBIT EARS* and *IPGA1* led to narrower petals in *Arabidopsi*s (Li et al., 2016; Yang et al., 2019). These studies suggested that flower petal development follows a three-stage process: fate determination, morphogenesis, and functional realization (Hepworth and Lenhard, 2014; Sablowski, 2015; Zhang et al., 2023), with petal morphogenesis being primarily regulated by cell number and size (Yamada et al., 2009; Prpic and Posnien, 2016; Walcher-Chevillet and Kramer, 2016). Therefore, research on variations related to petal shape, such as broading, narrowing, rounding, and shortening, remains to be explored.

This study aims to identify the key regulatory genes involved in the morphogenesis of lotus petal shape by examining the broad petal M512 and the narrow petal CSFY, alongside their respective wild-type. The findings will contribute to the development of techniques for artificially regulating lotus petal shapes, molecular marker-assisted breeding of novel lotus cultivars with unique petal shapes, and provide the background data for further exploration of the mechanisms underlying lotus petal formation.

## 2 Materials and methods

### 2.1 Plant materials

**Figure 1:**
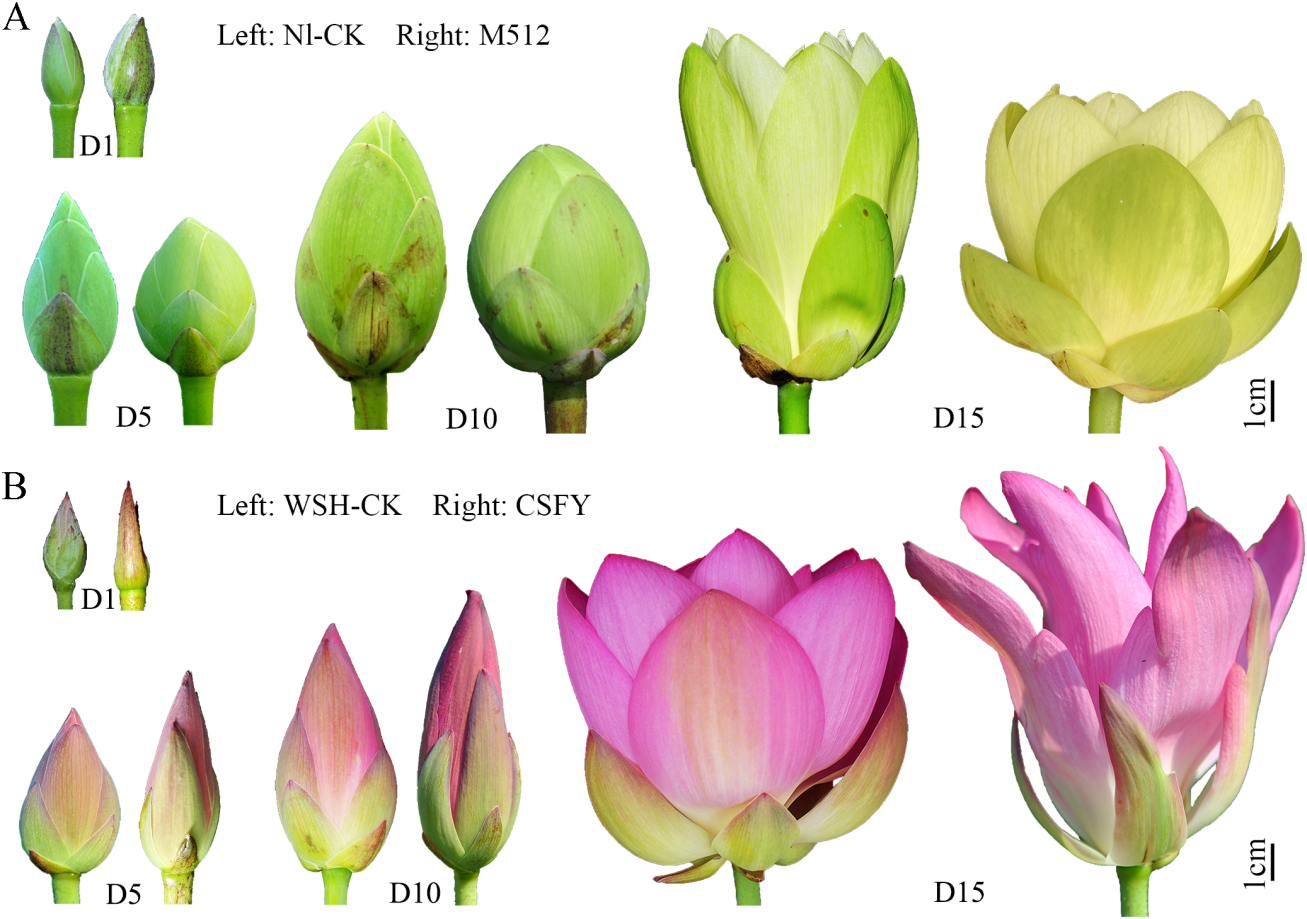
Morphological characteristics of floral buds (flowers) of the M512 vs. Nl-CK group **(A)** and the CSFY vs. WSH-CK group **(B)** at the 4 stages. D1, D5, D10 referred to day 1, day 5/6, and day 9/10 after the flower bud emerged from the water, respectively, and D15 indicated the first day of flowering.

Two variants of lotus petal shape and their respective wild types were studied (**Figure 1**; **Table 1**): (1) The broad-petaled American lotus variant M512, derived from a natural variation of a seedling of wild-type *N. lutea* (Nl-CK) (Liu et al., 2020). (2) The narrow-petaled Asian lotus variant ‘Chenshan Feiyan’ (CSFY), developed through ^60^Co γ-ray radiation of seeds from the wild-type *N. nucifera* Weishan Hong (WSH-CK) (Liu et al., 2021). All materials were cultivated at the International Nelumbo Collection in Shanghai Chenshan Botanical Garden.

**Table 1:** Basic information of the 4 lotus samples and the sampling standards for the 4 bud stages.

Plant code	Sample name	Sample origin	Species	Petal Shape	Petal number per flower	Bud stage and bud height/cm			
						D1^1^	D5	D10	D15
M512	M512	wild	*N. lutea*	broad	21–24	2.0 ± 0.2	4.0 ± 0.3	5.5–6.0	The first day of bloom
Nl-CK	American lotus	wild	*N. lutea*	normal	22–24	2.0 ± 0.2	4.5 ± 0.3	7.0–7.5	The first day of bloom
CSFY	‘Chenshan Feiyan’	cultivar	*N. nucifera*	narrow	18–22	2.0 ± 0.2	5.0 ± 0.3	7.5–8.2	The first day of bloom
WSH-CK	Weishan Hong	wild	*N. nucifera*	normal	22–25	2.0 ± 0.2	4.5 ± 0.3	7.0–7.5	The first day of bloom

^1^ D1, D5, and D10 represented the 1st day, 5th/6th day, and 9th/10th day after the flower bud emerged from the water, respectively, and D15 was to be the first day of flowering.

The flower buds’ height and their pollen development process (Zhang et al., 2019) were considered to select 4 sampling times: Day 1, Day 5/6, Day 9/10, and the day of flowering (Day 14–16) (**Figure 1**; **Table 1**), for petal shape phenotyping and transcriptome analysis. Single flowers of lotus typically have 22–24 petals per flower, with the 8th–17th petals (from outside to inside, located in the middle portion of the flower) being the most stable and representative in morphology (**Supplementary Figures S1, S2**). Thus, all samples in this study were collected from petals 8th to 17th.

### 2.2 Measurement of petal shape and epidermal cell density

In the broad-petaled M512 and Nl-CK, narrow-petaled CSFY and WSH-CK at stages D1, D5, D10, and D15, the length and width of the middle layer petals (8/9th to 16/17th) were measured under maximum stretch, and the length-to-width ratio was calculated. Differences in these 3 morphological indices were compared between variants and their wild types. Three flower buds (flowers) were selected for each stage as replicates.

According to the actual bud development, 3 petals (e.g., 8th, 11th, 14th or 9th, 12th, 15^th^, see **Supplementary Figures S1, S2**) were chosen at intervals of 2–3 petals to observe epidermal cell density. Five observation points were selected on the inner surface of each petal: the upper, middle, and lower quartile points along the longitudinal axis of the petal, with the middle point located on the horizontal line at the widest part of the petal. Then, on this widest horizontal line, left and right sites at the quarter division were picked (**Supplementary Figure S3**). At each site, epidermal cells were imprinted using the nail polish imprint method (Van Es et al., 2018) on a 0.5 cm × 0.5 cm petal area. An optical microscope (Zeiss Axio Imager 2 Pol) at 40× magnification collected 2 field views of epidermal cell at each site. The number of epidermal cell in each field view was manually counted using ImageJ software (Collins, 2007), with the cell count per field representing the cell density at that petal site.

### 2.3 RNA sequencing

Three buds (flowers) were collected per stage for M512 and Nl-CK, CSFY and WSH-CK. According to epidermal cell observation methods, 3 petals in the middle layer of each bud were collected, removing parts outside the top and bottom sites that were 2 of the 4 equidistant points located on the longitudinal axis of the petal. Then the remaining petal tissue from 3 floral buds (9 petals in total) was pooled to form one RNA extraction sample. Three biological replicates were prepared for each stage. Samples were immediately frozen in liquid nitrogen and stored at -80°C.

Total RNA was extracted from the tissue using TRIzol^®^ Reagent following the manufacturer’s instructions. RNA purification, reverse transcription, library construction, and sequencing were conducted at Shanghai Majorbio Bio-pharm Biotechnology Co., Ltd. The lotus petal RNA-seq transcriptome library was prepared using 1 μg of total RNA with the Illumina^®^ Stranded mRNA Prep, Ligation (San Diego, CA). After quantification with Qubit 4.0, sequencing was performed on the NovaSeq 6000 platform (PE150) using the NovaSeq Reagent Kit.

The raw paired-end reads were trimmed and quality controlled using Fastp (Chen et al., 2018) with default parameters. Clean reads were aligned to the lotus genomes (https://www.ncbi.nlm.nih.gov/genome/browse#!/eukaryotes/14095/) using HISAT2 (Kim et al., 2015) in orientation mode. The mapped reads of each sample were assembled by StringTie (Pertea et al., 2015) in a reference-based approach. The clean reads generated from each sample in the 4 stages within each group, along with their mapping efficiencies on the reference genome, were provided in **Supplementary Table S2**.

### 2.4 Differentially expressed genes filtering and time-course DEGs analysis

To identify differentially expressed genes (DEGs) between M512 and Nl-CK, between CSFY and WSH-CK at each flower bud stage, the expression level of each transcript was calculated using the transcripts per million reads (TPM) method. Gene abundances were quantified with RSEM (Li and Dewey, 2011). Differential expression analysis was conducted using DESeq2 (Love et al., 2014). DEGs with |log2FC| ≥ 1 and Padjust (FDR) < 0.05 were considered significantly differentially expressed. Using the R package maSigPro (http://www.bioconductor.org/packages/release/bioc/html/maSigPro.html), Time-course DEGs was performed on the DEGs obtained from the 2 groups at D1, D5, D10, and D15, resulting in the selection of a significant gene cluster (*P* < 0.05).

### 2.5 Identification of key genes for the development of broad and narrow petals

Within each group, firstly, gene ontology (GO) and kyoto encyclopedia of genes and genomes (KEGG) enrichment analysis were performed by TBtools-II (Chen et al., 2023), Goatools (Klopfenstein et al., 2018) and the Python scipy package (Virtanen et al., 2020) on the up-regulated genes (URGs) and down-regulated genes (DRGs) at 4 stages, respectively. Only entries with Padjust < 0.05 and the corresponding DEGs were retained for further analysis. Secondly, we removed DEGs that showed no significant differences (Padjust < 0.05) at both D1 and D5 stages. Next, the following 3 categories of DEGs will be further excluded: DEGs with expression value lower than 1.0 across D1, D5, and D10 stages; DEGs that appeared in at least 2 abiotic-related (e.g., stress resistance) GO/KEGG entries; and DEGs with functions clearly unrelated to morphological development, as indicated by previous studies. The DEGs remaining after these 5 filtering steps were considered candidate genes associated with the development of broad and narrow petal shapes in lotus.

### 2.6 Real-time quantitative PCR to validate the results of RNA-sequencing

To validate the accuracy of the RNA-sequencing of the 2 groups of lotus petals, 3 DEGs were randomly selected from each group for Real-time quantitative PCR (RT-qPCR) analysis at stages D1 and D10 (**Supplementary Table S1**). Using the PrimeScript™ RT reagent Kit with gDNA Eraser (Takara), RNA samples were reverse transcribed into cDNA. Subsequently, using cDNA as a template, with *β-NnACTIN* as the reference gene, quantitative detection was conducted on an Applied Biosystems StepOnePlus Real-Time PCR Machine. The reaction was conducted following the instructions of the TB Green^®^ Premix Ex Taq™ (Tli RNaseH Plus) kit (Takara). Each sample underwent 3 technical replicates, and the relative expression of DEGs was calculated using the 2^ΔΔCt^ method.

## 3 Results

### 3.1 Variation in petal length, width, and length-to-width ratio of lotus with broad/narrow petals

**Figure 2:**
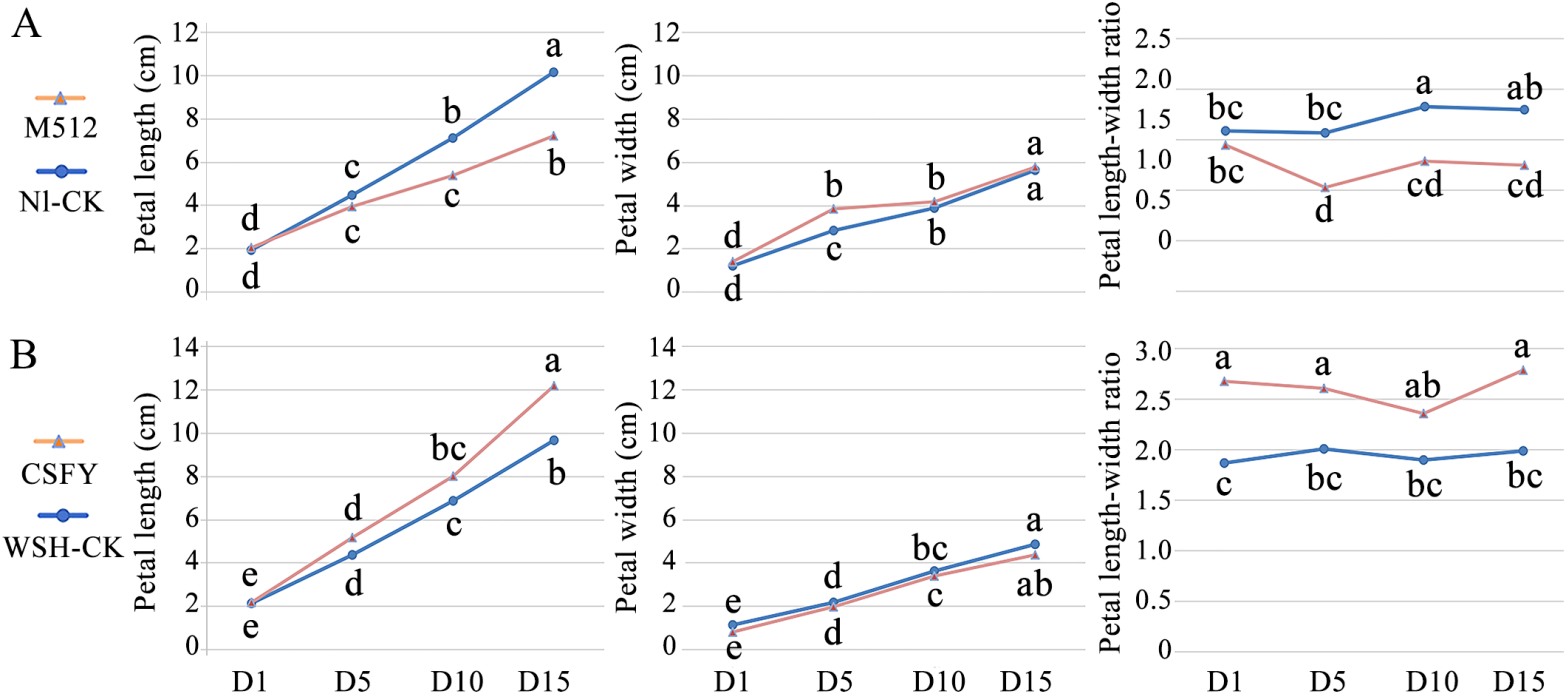
Morphological change trends of variant and normal petals across 4 floral bud stages. **(A)** The broad petal variant M512 vs. the American lotus Nl-CK. **(B)** The narrow petal variant CSFY vs. the Asian lotus WSH-CK. Lowercase letters indicated significant differences at *P*<0.05 level.

A comparison of morphological variations between the broad-petal variant M512 and the normal Nl-CK revealed that the average petal length of the middle petals (from the 8th/9th to the 15th/16th) in M512 was significantly shorter than in Nl-CK at later stages (D10 and D15) (**Figure 2A**). The petal width of M512 was notably greater than that of Nl-CK only at D5, while no striking difference was observed at the other 3 stages. However, the length-to-width ratios of M512 petals were significantly lower than those of Nl-CK at D5, D10, and D15. These results suggest that the broad-petal variation in M512 is primarily due to a reduction in petal length rather than an increase in width (**Figure 2A**, **Supplementary Figure S1**), and that the critical transition in broad-petal morphology occurs between D1 and D5 (**Figure 2A**).

In the narrow CSFY vs. WSH-CK group, petal length in CSFY was generally greater than in WSH-CK during the 4 bud stages, with a significant difference only at D15. The petal width showed no significant difference between CSFY and WSH-CK (**Figure 2B**), despite visible narrowing at the base of CSFY petals (**Supplementary Figure S2A**). However, CSFY displayed significantly higher length-to-width ratios compared to WSH-CK at D1, D5, and D15. Based on these findings, along with the observed petal morphology (**Supplementary Figure S2**), we propose that the narrowing of CSFY petals is achieved through a coordination of increased petal length and reduced petal width, with the fate of this variation likely determined before D1.

### 3.2 Differences in epidermal cell density in broad/narrow petals

**Figure 3:**
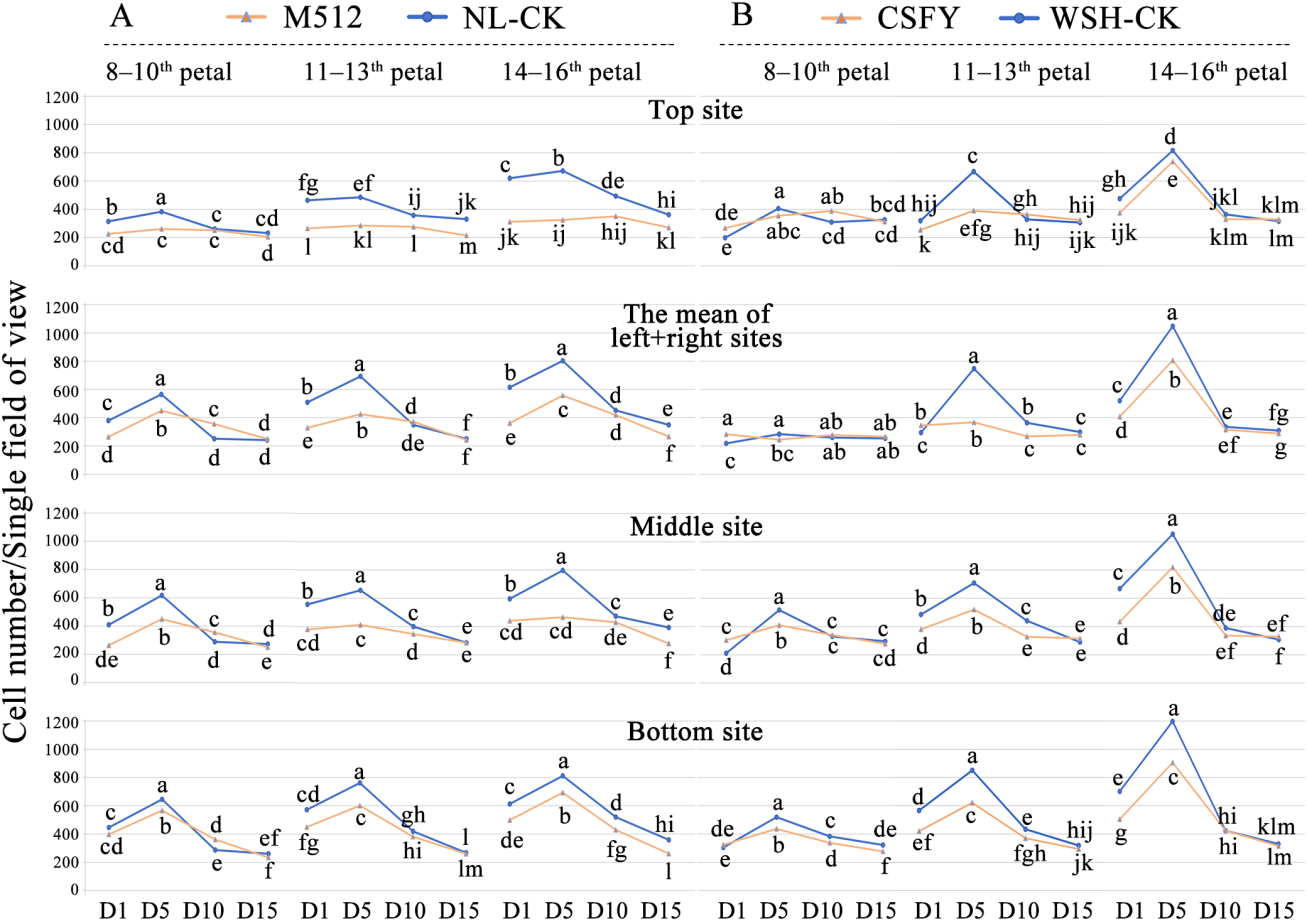
Changes and differences in epidermal cell density between variant and normal petals across 4 floral bud stages. **(A)** The broad petal variant M512 vs. the American lotus Nl-CK. **(B)** The narrow petal variant CSFY vs. the Asian lotus WSH-CK. Lowercase letters indicated significant differences (*P*<0.05) in cell density of each site on the petals among the 4 stages.

Analysis of epidermal cell density across 4 developmental stages revealed that in both groups, the overall cell density at 4 sites on the petal (top, the mean of left and right, middle, and bottom, see **Supplementary Figure S3**) followed a trend of D5 > D1 ≥ D10 ≈ D15 (**Figure 3**). Coupled with microscopic observations of epidermal cells (**Supplementary Figures S4, S5**), we hypothesize that D1 to D5 represents the rapid division phase, characterized predominantly by cell proliferation, as evidenced by many epidermal cells actively dividing or recently completed division during this stage. From D5 to D10, cell expansion predominates, reducing cell density due to increased cell volume. By D10 to D15, petal development approaches maturity, reflected in fewer cells in division and smaller fluctuations in cell density. Furthermore, a clearer trend in epidermal cell density was observed from outer to inner petals (**Figure 3**).

Compared to normal petals, except for the outer (8th-10th) petals of CSFY vs. WSH-CK, the epidermal cell density of the variant petals (both round M512 and narrow CSFY) was significantly lower at early stages (D1 and D5), particularly at D5 (**Figure 3**). As the flower buds developed, the epidermal cell density of M512 and CSFY gradually approached that of their respective normal samples by D10, with no stark differences by D15. This suggests that, by the time the flowers blooming (D15), the size of epidermal cells in both wide (M512) and narrow (CSFY) petals was nearly consistent with that of normal petals. Furthermore, microscopic observations of epidermal cells across the 4 bud stages (**Supplementary Figures S4, S5**) revealed no dramatic changes in cell size (e.g., enlargement or reduction) or cell shape (e.g., elongation or widening) between the variant samples and their controls. Therefore, it is inferred that the round M512 and narrow CSFY variations are primarily determined by the number of petal cells and their arrangement in the longitudinal and transverse dimensions.

Coupled with the fact that “the length of M512 petals decreased” and “the length and width of CSFY petals did not show significant changes, but the length-to-width ratio differed significantly,” we conclude that the broad development of M512 petals is mainly due to fewer cell divisions in the longitudinal direction (length) between D1 and D5, leading to an overall reduction in cell number. The narrow development of CSFY petals is achieved through coordinated increases and decreases in cell numbers longitudinally (length) and transversely (width), respectively, during the D1 to D5 period. Therefore, D1 and D5 are crucial stages for identifying key regulatory genes involved in the morphological construction of the 2 petal shapes.

### 3.3 The number and time-course analysis of DEGs in broad/narrow petals

**Figure 4:**
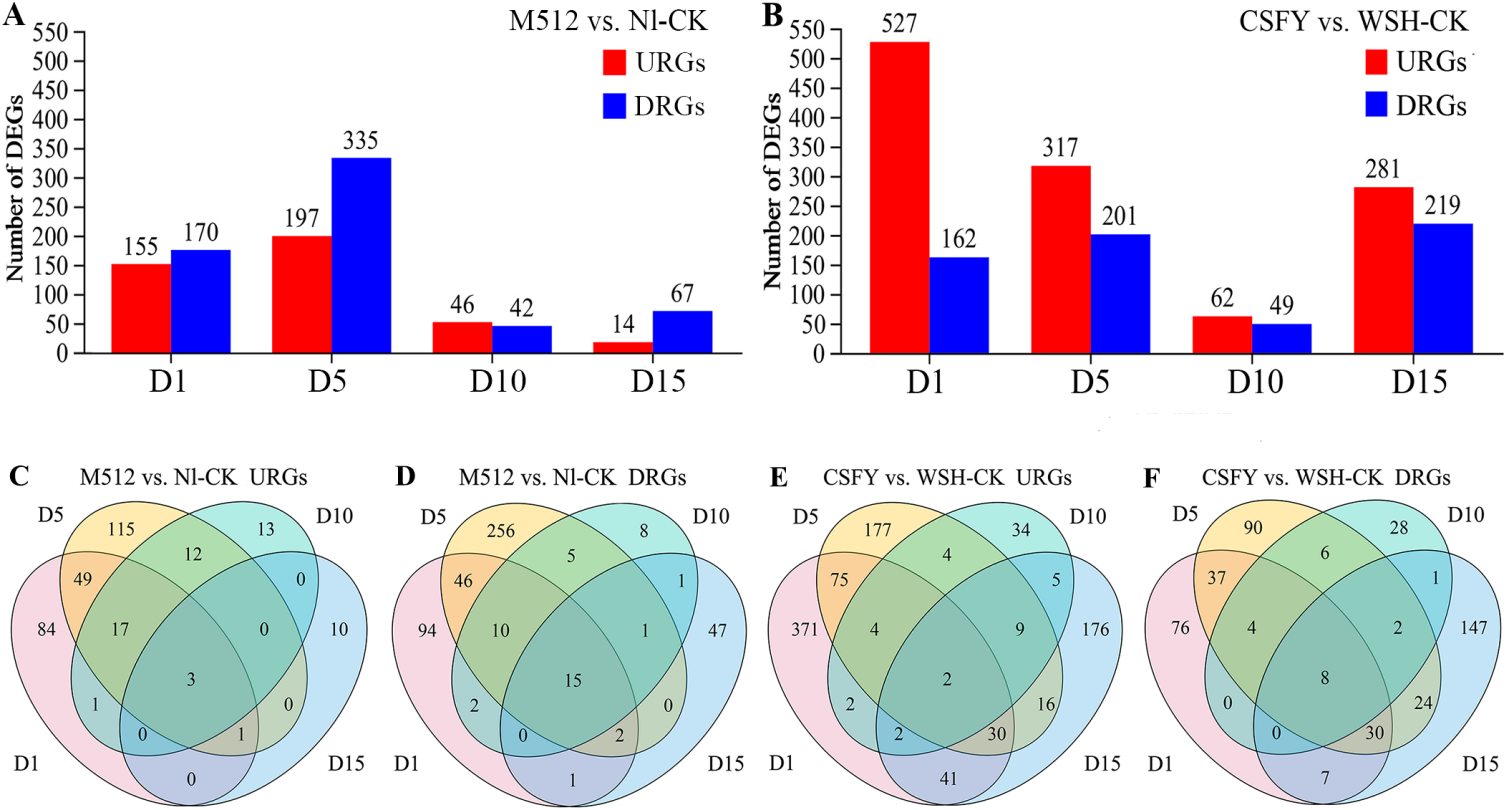
The URGs and DRGs numbers in the M512 vs. Nl-CK group **(A, C, D)** and the CSFY vs. WSH-CK group **(B, E, F)** at the 4 bud stages. URGs, up-regulated genes; DRGs, down-regulated genes.

Among the 4 stages of flower buds, there were 785 DEGs between the round M512 and Nl-CK in the American lotus group, including 305 URGs and 488DRGs (**Figure 4A**, **Supplementary Table S3**). In the Asian lotus group, there were 1,401 DEGs between the narrow CSFY and WSH-CK, with 948 URGs and 460 DRGs (**Figure 4B**, **Supplementary Table S4**). At stage D1 in the group of M512 vs. Nl-CK, it was found 6 members of the *ABC transporter G family* involved in the transmembrane transport of plant hormones, 5 *cytochrome P450* members involved in the synthesis and metabolism of plant hormones, as well as 5 *transcription factors*. At stage D5, a greater number of *ABC transporter G family* members and *cytochrome P450* genes were detected compared to D1. Notably, 5 DnaJ proteins, uniquely present at this stage, are known to be involved in the regulation of plant organ development and signal transduction. Moreover, genes associated with cell wall biosynthesis, such as 3 proteins of xyloglucan endotransglucosylase/hydrolase (No.6, 241, and 327), were also identified (**Supplementary Table S3**). Across all 4 stages of M512 vs. Nl-CK, 3 URGs and 15 DRGs were shared (**Figures 4C, D**). Among the 3 URGs, only gene No.232 *vicilin-like antimicrobial peptides 2-2* has been annotated, and it primarily participates in plant defense responses. Of the 15 DRGs, 8 were associated with the GO term “integral component of membrane”, while 7 were linked to GO terms related to various enzymatic activities. Additionally, 4 DEGs were assigned to specific pathways, including “ubiquitin mediated proteolysis/protein processing in endoplasmic reticulum”, “Tryptophan metabolism”, phenylpropanoid biosynthesis, and “ribosome” (**Supplementary Table S3**).

In the group of CSFY vs. WSH-CK,14 *cytochrome P450* members were identified at D1 stage—more than in the M512 vs. Nl-CK group. Among these, No.272, 419, 631, 656 were also present at D5 stage. Furthermore, 12 *ethylene-responsive transcription factors*, which play crucial roles in organ development, senescence, and hormone coordination, were detected at D1 stage; of these, No.80, 179, and 340 also exhibited significant differential expression at D5 stage. Additionally, 15 *transcription factors* were identified at D1 stage, among which No.2, 82, 290, and 555 were also present at D5 stage (**Supplementary Table S4**). Two URGs were common to all 4 stages (**Figure 4E**): No.279 uncharacterized *LOC104597722* and No.669 *glucan endo-1,3-beta-glucosidase-like*, the latter of which is implicated in “carbohydrate transport and metabolism”. Eight DRGs were consistently present across all 4 stages (**Figure 4F**). These genes were either involved in the “plant-type cell wall cellulose biosynthetic process”, related to the “integral component of membrane”, or participated in the “fatty acid biosynthetic process” (**Supplementary Table S4**).

**Figure 5:**
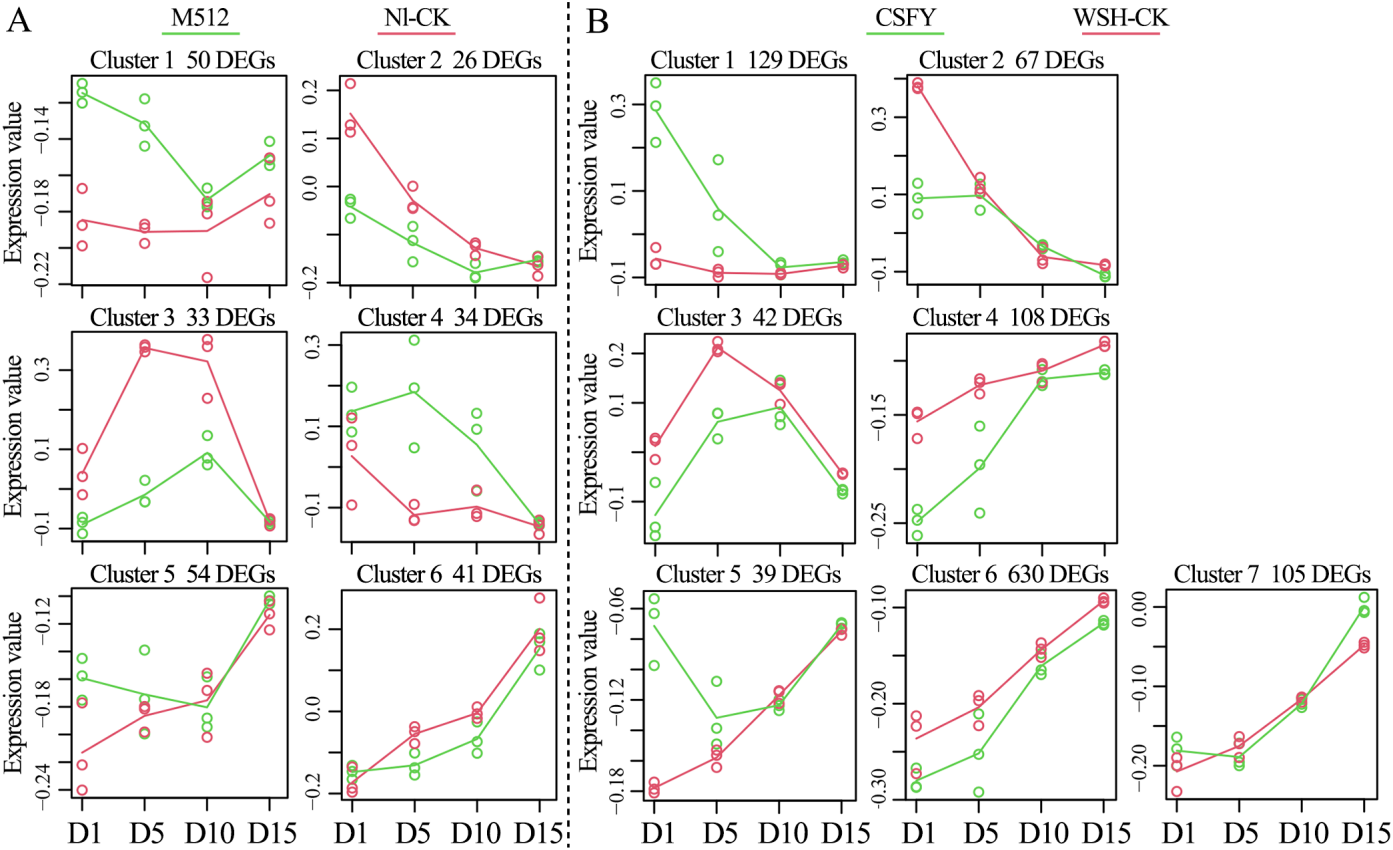
Temporal expression differences of DEGs in the broad petal group **(A)** and the narrow petal group **(B)** across 4 floral bud stages.

A time-course analysis of the DEGs enables the identification of not only when genes are differentially expressed but also how they change. In the M512 vs. Nl-CK group, 238 DEGs were categorized into 6 clusters with distinct expression trends. DEGs in clusters 1 and 2 showed significant expression changes at D1 and D5, while that in clusters 3 and 4 exhibited prominent differences at D5 and D10 (**Figure 5A**, **Supplementary Table S3**). Notably, 32% of DEGs in cluster 1 and 27% in cluster 2 were associated with the GO term “integral component of membrane”, which plays a crucial role in substance transport, signal transduction, and the maintenance of cellular structural homeostasis in plants. Moreover, 20% of cluster 1 DEGs were found within the pathway of “protein processing in endoplasmic reticulum”, which is responsible for the entire chain of protein modification, folding, and secretion. DEGs in cluster 3 were enriched in a broad array of GO terms lacking a dominant functional theme. In contrast, 38% of DEGs in cluster 4 encode heat shock proteins, and 12 DEGs were also assigned into the “protein processing in endoplasmic reticulum” pathway (**Supplementary Table S3**).

In the CSFY vs. WSH-CK group, 1,120 DEGs were classified into 7 temporal expression clusters. DEGs in clusters 1, 3, and 4 exhibited significant changes at stages D1 and D5, while clusters 2 and 5 showed significant differences only at stage D1 (**Figure 5B**, **Supplementary Table S4**). The most enriched GO term in clusters 1, 3, and 4 was “integral component of membrane”, with corresponding DEGs comprising 22%, 40%, and 42% of each cluster, respectively, paralleling findings in clusters 1 and 2 from the M512 vs. Nl-CK group. In cluster 1, enriched pathways included “plant hormone signal transduction”, “pentose and glucuronate interconversions”, “MAPK signaling pathway”, and “amino sugar and nucleotide sugar metabolism”, all of which are associated with cell wall biosynthesis, secondary metabolite modification, and hormone signaling. Clusters 3 and 4 were mainly involved in pathways such as “cutin, suberine and wax biosynthesis”, “fatty acid elongation”, and “brassinosteroid biosynthesis”. In Cluster 2, 11 DEGs were linked to the GO term “oxidoreductase activity”, and 24 DEGs were mapped to pathways predominantly related to “ascorbate and aldarate metabolism”, “inositol phosphate metabolism”, and “cutin, suberine and wax biosynthesis”. Cluster 5 featured 7 DEGs enriched in the GO term “transcription factor activity, sequence-specific DNA binding,” though only 5 DEGs were classified into pathways such as “plant hormone signal transduction” and “protein processing in endoplasmic reticulum” (**Supplementary Table S4**).

Thus, the trends in DEG numbers and their temporal expression patterns across the 4 stages (**Figures 4**, **5**) align with the hypothesis derived from petal shape metrics, epidermal cell density, and microscopic observations that “the development of round and narrow petal morphologies is primarily determined during the early stages of D1 and D5.” Consequently, the next step will be to identify key genes involved in the morphological construction of broad and narrow petals among the DEGs that exhibited significant differences in expression values or expression patterns during D1 and D5.

### 3.4 GO and KEGG enrichment analysis of DEGs associated with petal broadness/narrowness variation in lotus

**Figure 6:**
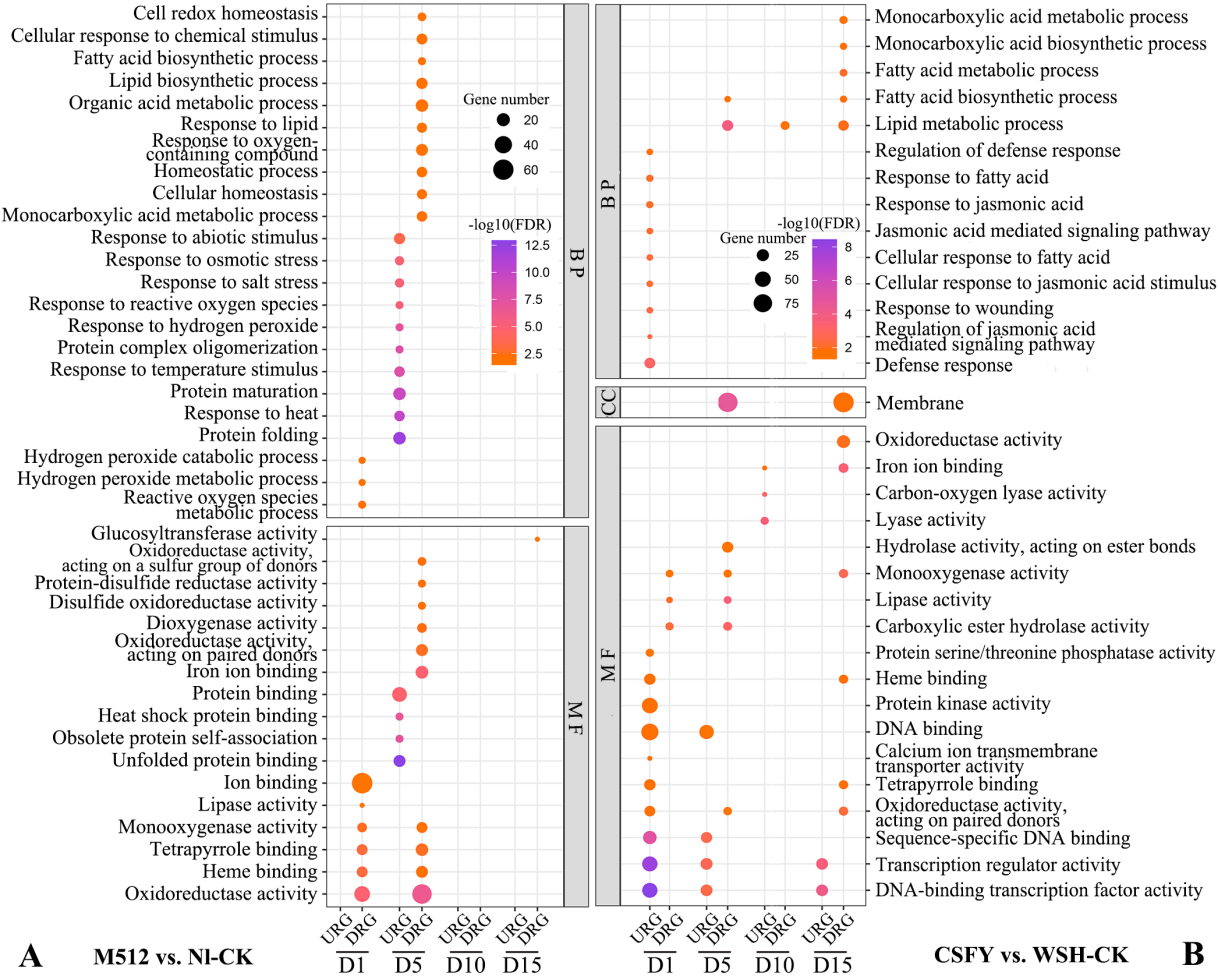
GO enrichment of DEGs from the broad petal group **(A)** and the narrow petal group **(B)** (Top 10 entries). BP, biological process; CC, cellular component; MF, molecular function; URGs, up-regulated genes; DRGs, down-regulated genes.

In groups of M512 vs. Nl-CK and CSFY vs. WSH-CK, 75 and 56 GO terms were enriched, respectively (**Supplementary Tables S5, S6**). In the M512 vs. Nl-CK group, the top 3 significantly enriched biological process (BP) terms were “protein folding”, “response to heat”, and “protein maturation”. In the molecular function (MF) category, the top 3 enriched terms were “unfolded protein binding”, “obsolete protein self-association”, and “heat shock protein binding” (**Figure 6A**; **Supplementary Table S5**). Protein synthesis and maturation (such as proper folding, glycosylation and phosphorylation) directly influence the activities of enzymes involved in cell wall and hormone biosynthesis, thereby regulating the direction and rate of cell expansion. Heat shock proteins can stabilize transcription factors (e.g. MADS-box proteins) or cytoskeletal proteins, in turn affecting the cell morphology during organ development.

In the CSFY vs. WSH-CK group, the top 3 BP terms were “defense response”, “regulation of jasmonic acid-mediated signaling pathway”, and “response to wounding”, while the top 3 MF terms were “DNA-binding transcription factor activity”, “transcription regulator activity”, and “sequence-specific DNA binding” (**Figure 6B**, **Supplementary Table S6**). Jasmonic acid (JA) may interact with auxin/ethylene signaling pathways to guide petal elongation or the morphology of petal margins. Transcription factors can directly regulate functional genes associated with organ shape development, such as those involved in cell expansion and polar growth. While DNA-binding transcription factors may modulate chromatin status or enhance the binding capacity of other transcription factors, thereby fine-tuning downstream gene expression to shape the organ. Sequence-specific DNA binding ensures a high degree of specificity and precision in target gene regulation, allowing key developmental genes to be expressed at the right time and in the correct spatial context. This precise regulation is critical for the natural formation of morphological traits.

Shared GO terms between the 2 groups included 2 BP categories (“monocarboxylic acid metabolic process” and “fatty acid biosynthetic process”) as well as 7 MF categories: “oxidoreductase activity”, “iron ion binding”, “monooxygenase activity”, “lipase activity”, “heme binding”, “tetrapyrrole binding”, and “oxidoreductase activity acting on paired donors” (**Figure 6**). Cell expansion in plant organs depends on membrane fluidity, which is influenced by the composition of fatty acids, such as the proportion of unsaturated fatty acids. Certain oxidoreductases regulate cell wall loosening or programmed cell death, while several monooxygenases are involved in maintaining the homeostasis of abscisic acid and brassinosteroids, thus controlling cell elongation.

**Figure 7:**
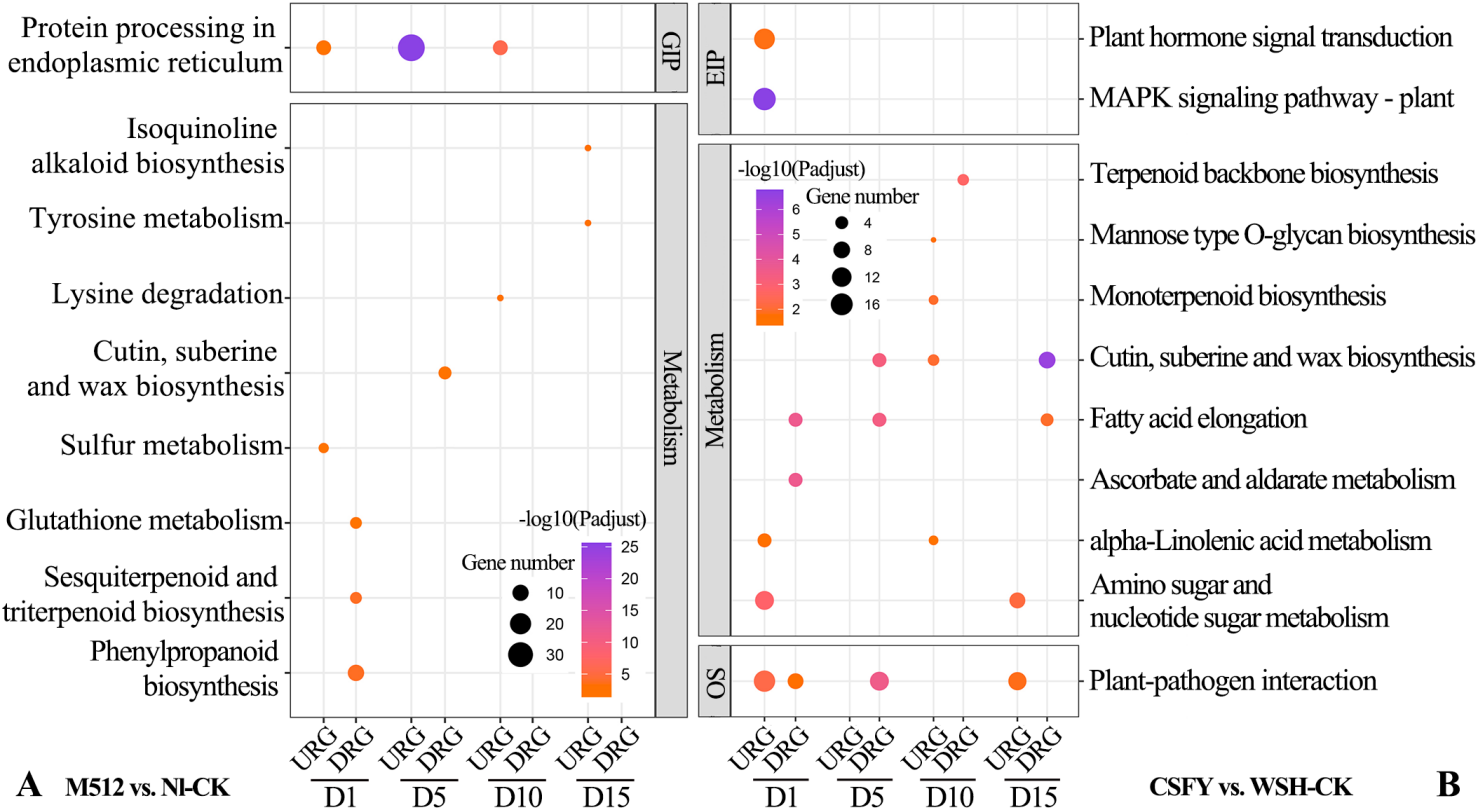
KEGG enrichment of DEGs from the broad petal group **(A)** and the narrow petal group **(B)**. GIP, genetic information processing; EIP, environmental information processing; OS, organismal systems. URGs, up-regulated genes; DRGs, down-regulated genes.

In the KEGG enrichment for M512 vs. Nl-CK, 9 pathways were identified (**Figure 7A**). Among them, “protein processing in the endoplasmic reticulum”, a pathway under genetic information processing (GIP), was the most significant and was consistently present during D1, D5, and D10 stages. In the metabolism category, enriched pathways included “phenylpropanoid biosynthesis”, “sesquiterpenoid and triterpenoid biosynthesis”, “tyrosine metabolism”, “isoquinoline alkaloid biosynthesis”, “lysine degradation”, “glutathione metabolism”, “sulfur metabolism”, and “cutin, suberine, and wax biosynthesis”. Among these pathways, phenylpropanoid biosynthesis is involved in lignin deposition, which strengthens the cell wall. Brassinosteroids, a type of triterpenoid, promote cell elongation through the brassinazole-resistant transcription factors. The ratio of reduced glutathione to oxidized glutathione modulates the intensity of reactive oxygen species signaling, influencing the activity of cell wall-loosening enzymes and thus determines the rate of cell expansion. And variations in cuticle thickness may restrict localized cell expansion.

For the CSFY vs. WSH-CK group, 11 KEGG pathways were enriched (**Figure 7B**). Under GIP category, “MAPK signaling pathway” exhibited the highest significance at the early D1 stage of floral bud development. In the metabolism category, 8 pathways were involved, including “cutin, suberine, and wax biosynthesis”, “ascorbate and aldarate metabolism”, “fatty acid elongation”, “amino sugar and nucleotide sugar metabolism (related to cell wall synthesis, plant hormone biosynthesis, and responses to environmental stress)”, “terpenoid backbone biosynthesis”, “monoterpenoid biosynthesis”, “mannose-type O-glycan biosynthesis”, and “alpha-linolenic acid metabolism”. Only 1 pathway, “cutin, suberine, and wax biosynthesis”, was shared between the American and Asian lotus groups. In those pathways, fatty acid elongation supplies long-chain fatty acids essential for the biosynthesis of plant cell membranes, ensuring their fluidity and stability, which is crucial for maintaining normal cellular morphology and function. The metabolism of amino sugars and nucleotide sugars provides precursors for cell wall polysaccharides, such as galacturonic acid and xylose, which directly adjust the rigidity and extensibility of the cell wall. Rapid cell expansion during petal development may rely on such metabolic products to remodel the structures of cell wall. While the synthesis and modification of the cell wall greatly affect plant cell elongation, differentiation, and arrangement, ultimately shaping the organs. In mannose-type O-glycan biosynthesis, O-glycan modifications impact the structure and function of secretory and membrane-bound proteins, thus also affecting plant cell growth, differentiation, and interactions, which collectively determine organ shape.

### 3.5 Key genes regulating broad/narrow petal morphologies in lotus

**Figure 8:**
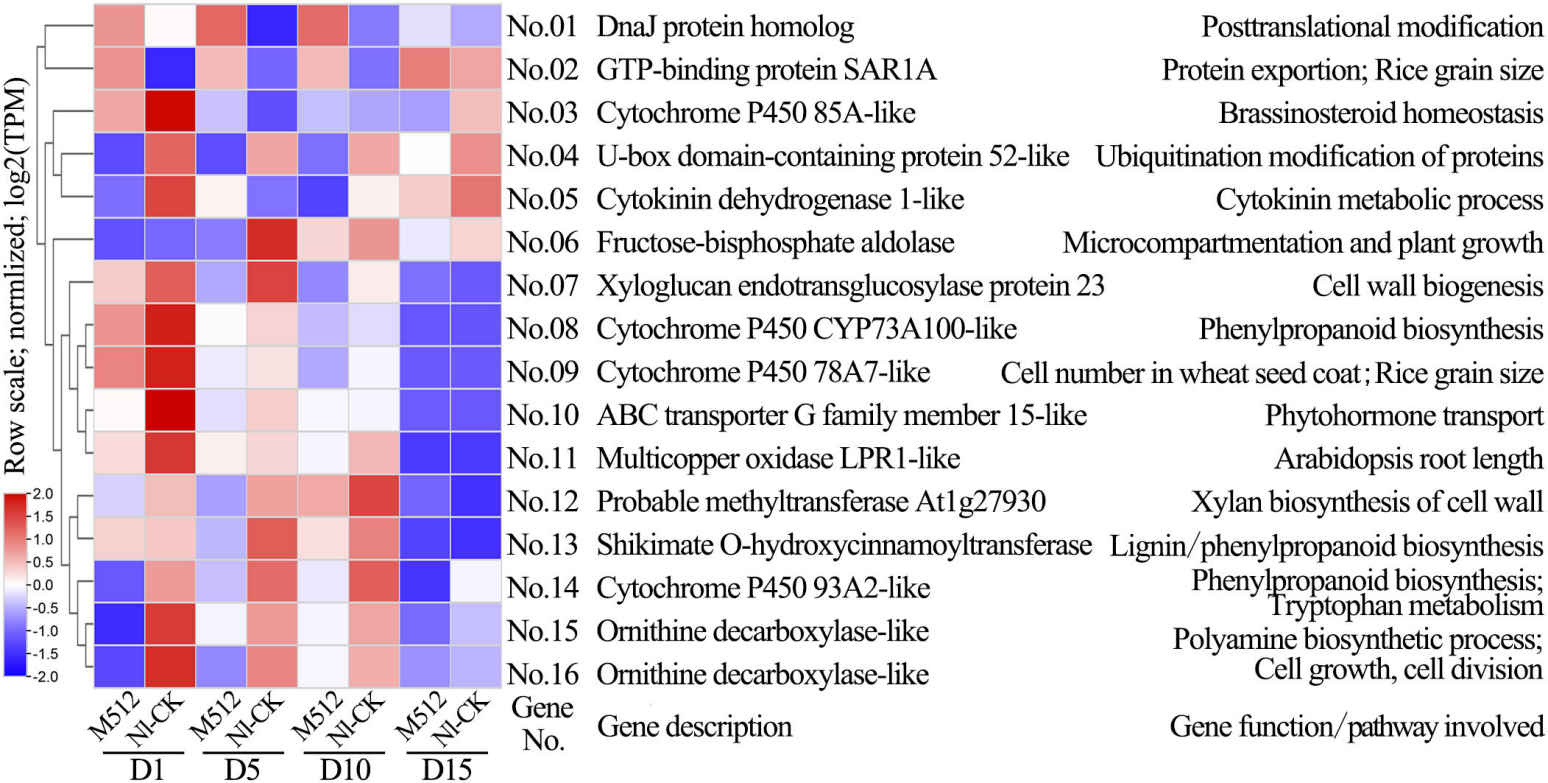
Clustering diagram of expression patterns and functions of partial candidate genes in the M512 vs. Nl-CK group. The genes codes represented by the gene No.s and the references exhibiting gene function/pathway were provided in **Supplementary Table S7**.

Based on a five-step screening process, key candidate genes involved in the regulation of broad and narrow petal development in lotus were determined. In M512 vs. Nl-CK, 59 candidate genes were identified, 26 of which belonged to clusters 1–4 (**Supplementary Tables S3, S7**; **Figure 5A**). Among these, 16 genes have been previously validated in other plants as being associated with processes such as cell development, hormone biosynthesis, or metabolism (**Figure 8**). No.09 *cytochrome P450 78A7-like* regulates cell number and rice grain size. No.15 and No.16 are both *ornithine decarboxylase-like* genes, participating in cell growth and division as well as auxin biosynthesis. No.07 *xyloglucan endotransglucosylase/hydrolase protein 23* and No.12 *methyltransferase At1g27930* directly regulate cell wall construction. No.08 *cytochrome P450 CYP73A100-like*, No.13 *shikimate O-hydroxycinnamoyltransferase-like*, and No.14 *cytochrome P450 93A2-like* are involved in the biosynthesis of cell wall components such as lignin. Several genes—No.03 *cytochrome P450 85A-like*, No.05 *cytokinin dehydrogenase 1-like*, No.10 *ABC transporter G family member 15-like*, No.14 *cytochrome P450 93A2-like*, No.15 and No.16 *ornithine decarboxylase-like*—are implicated in hormone signaling pathways, including cytokinin, auxin, and brassinosteroids. Among these 16 candidate genes, No.01 D*naJ protein homolog* and No.02 *GTP-binding protein SAR1A* exhibited consistently up-regulated expression across all 4 floral bud stages in the broad M512, compared to the normal Nl-CK. In contrast, the expression levels of 14 genes (No.03–No.16) were predominantly down-regulated during stages D1, D5, and D10 in M512 (**Figure 8**, **Supplementary Table S7**).

**Figure 9:**
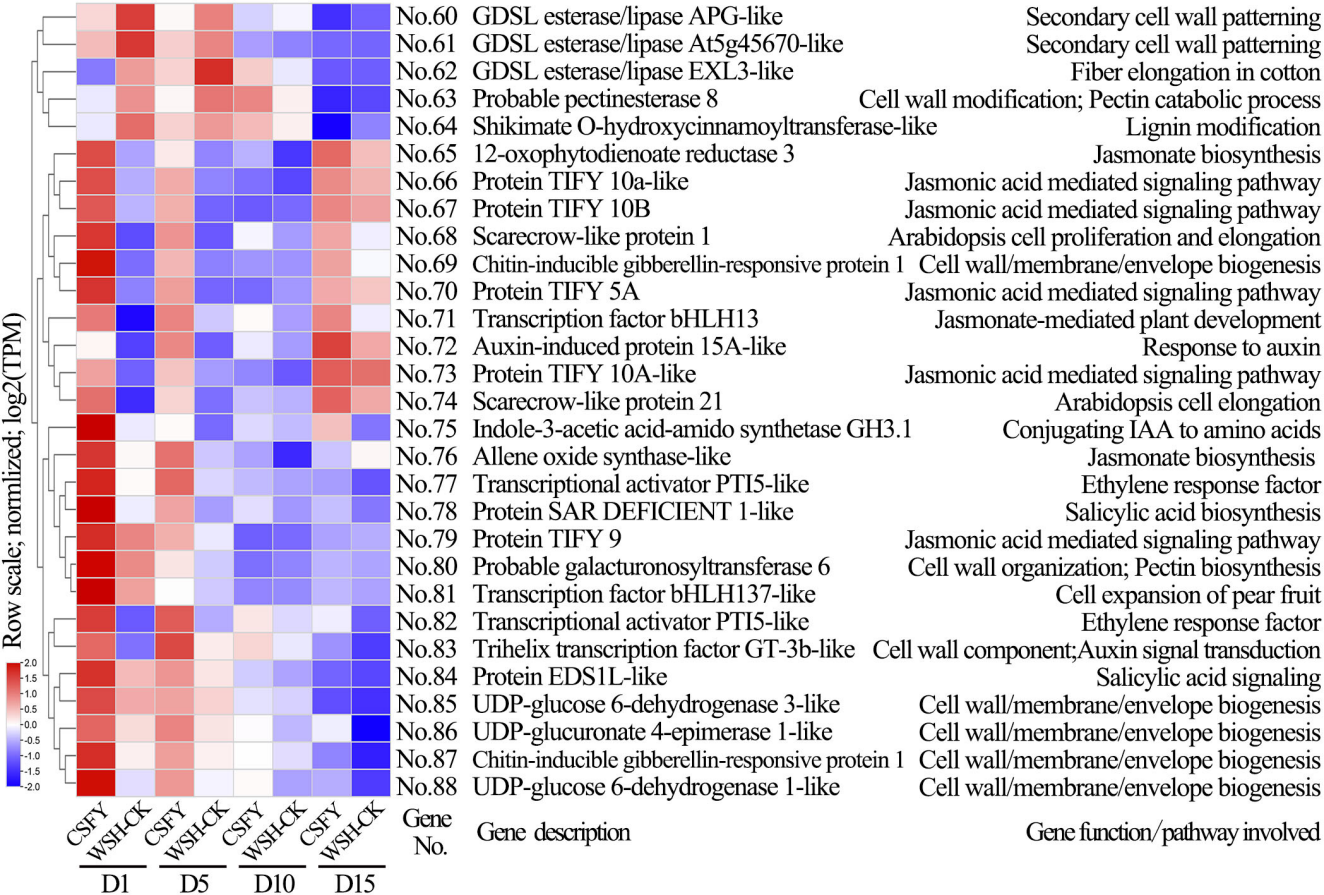
Clustering diagram of expression patterns and functions of partial candidate genes in the CSFY vs. WSH-CK group. The genes codes represented by the gene No.s and the references exhibiting gene function/pathway were provided in **Supplementary Table S7**.

In the CSFY vs. WSH-CK group, 96 candidate genes were identified for narrow petal variation, of which 71 overlapped with DEGs of clusters 1–7 (**Supplementary Tables S4, S7**; **Figure 5B**). Of these, 29 were linked to cell development and hormone signaling pathways (**Figure 9**). These 29 genes showed significant differential expression between CSFY and WSH-CK at early stages D1 and D5, with the majority (24 genes, No.64–No.87) up-regulated and a minority (5 genes, No.60–No.64) down-regulated in narrow CSFY compared to the controls. These genes primarily function in cell wall (membrane) morphogenesis, cell division, expansion, and elongation (No.62 *GDSL esterase/lipase EXL3-like*, No.68 *scarecrow-like protein 1*, No.74 *scarecrow-like protein 21*, No.81 *transcription factor bHLH137-like*), jasmonic acid-mediated signaling (No.66 *TIFY 10a-like*, No.67 *TIFY 10B*, No.70 *TIFY 5A*, No.73 *TIFY 10A-like*, No.79 *TIFY 9*), and hormone signaling pathways including auxins and ethylene (No. 72 *auxin-induced protein 15A-like*, No. 75 *indole-3-acetic acid-amido synthetase GH3.1*, No. 77=No.82 *pathogenesis-related genes transcriptional activator PTI5-like*, and No. 83 *trihelix transcription factor GT-3b-like*).

In the American lotus group (M512 vs. Nl-CK), 87.5% (14/16) of key candidate genes were significantly down-regulated in the variant M512, whereas in the Asian lotus group (CSFY vs. WSH-CK), 82.8% (24/29) of key candidate genes were significantly up-regulated in the variant CSFY. Notably, only 2 shared genes were identified between the 2 groups: No.13=No.64 *shikimate O-hydroxycinnamoyltransferase-like*, involved in lignin biosynthesis, and No.55=No.130 probable *inositol oxygenase*, potentially contributes to the biosynthesis of nucleotide sugar precursors for cell-wall matrix polysaccharides (Kanter et al., 2005).

### 3.6 Validation of DEGs expression values via RT-qPCR

As most DEGs exhibited the largest differences in expression value between variant and control samples at the D1 and D5 stages, while the differences were smaller at D10 and D15, 6 randomly selected DEGs were subjected to RT-qPCR validation at the D1 and D10 stages. The results showed that the expression trends of the 6 DEGs were highly consistent with those observed in RNA sequencing (**Supplementary Figure S6**), confirming the reliability of RNA-seq data used for this study’s analyses.

## 4 Discussion

Based on the findings of this study, we propose that the development of broad and narrow petals in lotus undergoes 3 distinct stages: fate determination, morphogenesis, and functional realization. The period selected in this study, spanning from the first day after the flower bud emerges from the water to the day of blooming, primarily corresponds to the morphogenesis phase of petal shaping. Within this period, the critical phase occurs between the first day (D1) and the fifth or sixth day (D5) after flower bud emerged from water, which determines the total number of cells contributing to petal length and/or width. Subsequently, from D5 to full bloom (D15), petal growth is primarily driven by cell expansion, leading to an increase in petal volume or size within the pre-established morphological framework.

### 4.1 Observation and quantification of epidermal cell shape, density and size in lotus petals

The morphology of plant organs is determined by both cell number and cell size, which are regulated by cell proliferation and cell expansion, respectively (Komaki and Sugimoto, 2012). To elucidate whether the variation in lotus petal shape is due to changes in cell number or alterations in cell shape (e.g., elongation or broadening), we employed the nail polish imprint method to microscopically observe the epidermal cells of 4 samples from 2 groups (**Supplementary Figures S4, S5**). Our investigation first confirmed that lotus petals follow a growth pattern characterized by early-stage cell proliferation followed by late-stage cell expansion, consistent with previous studies (Szécsi et al., 2006; Yamada et al., 2009; Prpic and Posnien, 2016; Jing et al., 2023). Second, we found that the epidermal cells of lotus petals at the 4 developmental stages, particularly in the early stages, exhibited irregular shapes (**Supplementary Figures S4, S5**), making it challenging to precisely measure cell size, specific cell shape, or the intercellular space. Consequently, we could only indirectly represent cell size by statistically analyzing the number of cells per microscopic field. However, it was evident that the epidermal cells of the broad M512 variant did not exhibit significant lateral expansion, nor did the epidermal cells of the narrow CSFY variant display noticeable longitudinal elongation. Moreover, it is worth noting that nail polish imprinting introduces some variability due to methodological limitations and operator-dependent factors affecting observed cellular morphology. To achieve more precise measurements of epidermal cell shape, future studies could employ scanning electron microscopy to capture high-resolution images of cell structures, combined with machine learning techniques to automatically extract and analyze phenotypic data across samples.

The study found that the broad-petaled M512 phenotype resulted from a decrease in petal length while maintaining a constant petal width at maturity. In contrast, the narrow-petaled CSFY phenotype was attributed to a slight but non-significant decrease in petal width accompanied by a slight but non-significant increase in petal length. Although these 2 variants exhibited opposing trends in petal shape development, their density dynamics of epidermal cell across the 4 bud stages followed a similar pattern: at the upper, the left and right, middle, and basal positions, cell density was lower than that of the control in the early stages but became converged with the control at the later stages (**Figure 3**). This raises an important question for future research: How does the broad-petaled M512 regulate a reduction in the total number of longitudinal (lengthwise) petal cells while maintaining a stable transverse (widthwise) cell count? Similarly, how does the narrow-petaled CSFY coordinate the increase in longitudinal cell count alongside a reduction in transverse cell count?

### 4.2 Regulatory patterns and origins of broad and narrow variations in lotus petal shape

In this study, it demonstrated that the broad variation in the petals of the American lotus M512 is primarily due to a shortening of petal length, while the narrowing of petals in the Asian lotus CSFY is achieved through a combination of increased length and decreased width (**Figure 2**). Moreover, the GO and KEGG enrichment pathways, as well as the candidate genes identified, differed markedly between the 2 variants (**Figures 6**–**9**). And in the KEGG enrichment analysis, only 1 pathway was shared between American and Asian lotus groups. Among key candidate genes, those in the American lotus group (M512 vs. Nl-CK) tended to be down-regulated in expression value in the broad variant, whereas those in the Asian lotus group (CSFY vs. WSH-CK) were generally up-regulated in the narrow variant (**Figures 8**, **9**), with only 2 genes common to both groups (**Supplementary Table S7**). This results may be attributed to the differences in both the inducing factors and the species-specific genetic backgrounds.

The M512 and Nl-CK samples from the American lotus group, representing 1 of only 2 species in the *Nelumbo* genus, were derived from wild-type American lotus seedlings collected in Florida, USA (Liu et al., 2020). Thus, M512 likely arose through natural variation following normal sexual reproduction. And its broad petal phenotype may be attributed to small genetic variations, such as few single nucleotide polymorphisms (SNPs) or short insertions/deletions (indels), or to minor epigenetic modifications such as DNA methylation or histone modification. In contrast, the Asian lotus group came from another species within the *Nelumbo* genus, and the narrow petal phenotype of CSFY was induced by ^60^Co γ-irradiation. This mutagenic treatment likely caused genetic alterations at the chromosome level, potentially affecting many functional genes or even chromosome segments. Supporting this, CSFY also displayed additional morphological abnormalities, including fin-shaped or curled leaves, a drastic reduction in pistil number, and decreased pollen fertility (Liu et al., 2021). Collectively, if there are differences in the molecular regulatory patterns between the broad-petaled M512 and narrow-petaled CSFY, it likely correlates with the distinct induction pathways of the 2 variants.

### 4.3 Functions of candidate genes associated with broad/narrow petal in lotus

In screening for regulatory genes involved in petal shape development, this study particularly focused on the changes and differences in gene expression levels during the D1 and D5 stages. During these stages, rapid cell division dominated lotus petal life activities, with cell expansion playing a secondary role. These processes involved a series of physiological and biochemical activities, including hormone biosynthesis and metabolism, signal transduction, enzymatic (protein) interactions, cytoskeletal construction, synthesis and degradation of cell walls and membranes, as well as nutrient and energy supply (Winship et al., 2010; Buchanan et al., 2015; Li and Li, 2019).

In the broad M512 and narrow CSFY petals, 16 and 29 genes directly related to those biological activities were identified, respectively (**Figures 8**, **9**, **Supplementary Table S7**). In the broad M512 variant, 4 *P450* genes were obtained: *cytochrome P450 78A7*, *85A*, *93A2*, and *CYP73A100* (No.03, 08, 09, and 14) (**Supplementary Table S7**). The *TaCYP78A3* gene affected seed coat cell proliferation, ultimately determining grain size in wheat (Ma et al., 2015). Overexpression of cytochrome *P450 78A5* (*KLU*/*CYP78A5*) in *Arabidopsis* promoted excessive growth of petals, sepals, and leaves by increasing cell number, whereas its mutants exhibited reduced floral organ size due to decreased cell numbers (Anastasiou et al., 2007). *CYP85A1*, *CYP85A2*, and *CYP85A3* have been shown to influence the growth in *Populus* tree and plant architecture in *Arabidopsis* (Kwon et al., 2005; Jin et al., 2017). Additionally, cytochrome *P450 93A2* participates in tryptophan metabolism, and tryptophan is a precursor for auxin biosynthesis (Zhao, 2012). Notably, the down-regulated candidate gene No. 05 *LOC104606255* encodes cytokinin dehydrogenase 1, which catalyzes the irreversible degradation of cytokinins (Kowalska et al., 2010; Niemann et al., 2018), and cytokinins are crucial for controlling plant cell division, growth, and development (Dabravolski and Isayenkov, 2021). However, the specific mechanisms of these genes in petal shape variation in lotus still require further exploration.

In the candidate genes for the narrow CSFY petal variation, 5 genes (No.66 *TIFY 10a*-*like*, No.67 *TIFY 10B*, No.70 *TIFY 5A*, No.73 *TIFY 10A*-like, No.79 *TIFY 9*) encode JAZ subfamily of the TIFY protein family. These genes interact with various transcription factors or proteins to participate in multiple hormone signaling pathways, including jasmonic acid, ethylene, auxin, gibberellin, and abscisic acid, thereby affecting processes such as floral organ formation, flowering time, and seed development (Fernández-Calvo et al., 2011; Pauwels and Goossens, 2011; Zhao et al., 2024). The chrysanthemum TIFY family gene CmJAZ1-like is down-regulated in expression value during petal elongation, and overexpression of this gene inhibited petal cell expansion, leading to reduced flower diameter and shortened petals (Guan et al., 2022). Candidate genes No. 68 *LOC104599573* and No. 74 *LOC104594202* encode scarecrow-like protein 1 (SCL1) and scarecrow-like protein 21 (SCL21), respectively. The SCL21 protein is involved in cortical development and gibberellin signaling transduction in plants (Cui and Benfey, 2009). During the early development of rose petals, RhSCL28 participates in promoting cell division within the cytokinin-regulated pathway, thereby increasing petal size (Jin et al., 2025). In addition, 16 candidate genes were found to be involved in the mitogen-activated protein kinase (MAPK) signaling pathway (**Supplementary Table S7**). It is an important cellular signal transduction pathway that governs processes such as cell proliferation, growth, differentiation, and metabolism (Seger and Krebs, 1995; Calderini et al., 1998; Ma and Yu, 2010; Zhang et al., 2024).

Since the currently reported regulatory genes in petal development primarily targeted the natural growth process from juvenile to mature and then to senescence (Hu et al., 2003; Crawford et al., 2004; Varaud et al., 2011; Guo et al., 2024), few studies have specifically focused on the trait of petal shape and primarily represent incidental findings (Elliott et al., 1996; Li et al., 2016; Ren et al., 2016; Yang et al., 2019). As a result, none of the genes previously reported overlap with the candidate genes identified in this study. Furthermore, our research focused solely on the morphological development stage of lotus petal broadening or narrowing, without investigating the earlier stage of fate determination. A comprehensive understanding of the functions of the candidate genes identified in this study, combined with single-cell sequencing technology and real-time imaging of cell development, will help unravel the molecular mechanisms underlying the fate determination of lotus petal shape.

## 5 Conclusions

During the development of lotus petals after the flower buds emerge from the water, the early stages are primarily driven by cell division, while the later stages are characterized by cell expansion. For the broad and narrow variations of petal shape in this study, the fate determination of both petal shapes occurs before the flower bud emerges from the water. The mature morphology of these 2 petal shapes is primarily governed by cell proliferation (i.e., cell number) along the longitudinal (length) and transverse (width) directions of the petals, rather than by the size or shape of the cells. A total of 59 and 96 candidate genes might be associated with petal shape development in broad-petaled and narrow-petaled variants, respectively. Many of these candidate genes are associated with the development of cell walls and membranes, which significantly correlates with the fact that the broad and narrow petal shapes of the lotus are dominated by cell proliferation. Based on this study, we are constructing separate F_2_ populations for the 2 groups of variants to conduct bulked segregant RNA-seq (BSR-seq) analysis on individuals with extreme petal shapes. Subsequently, the differences in expression values of the narrowed-down candidate genes between the variants and the wild types will be assessed using RT-qPCR technology. Then genes exhibiting substantial differences in expression levels will be selected for further functional validation through experiments such as overexpression and gene knockout.

## Data Availability

The raw sequencing data have been deposited in the CNCB dataset (China National Center for Bioinformation) under the BioProject accession CRA025554 (https://ngdc.cncb.ac.cn/gsa). Further inquiries can be directed to the corresponding author.
