# Supplementary material for: Comparative transcriptomics of two petal variants reveals key functional genes underlying petal shape development in lotus (Nelumbo)
Source: Front Plant Sci. 2025 Jul 8;16:1596925. doi: 10.3389/fpls.2025.1596925 (PMC12279870; doi:10.3389/fpls.2025.1596925)
Supplement: Supplementary file 1 [file DataSheet1.zip › Supplementary Figure S1–S6.PDF]

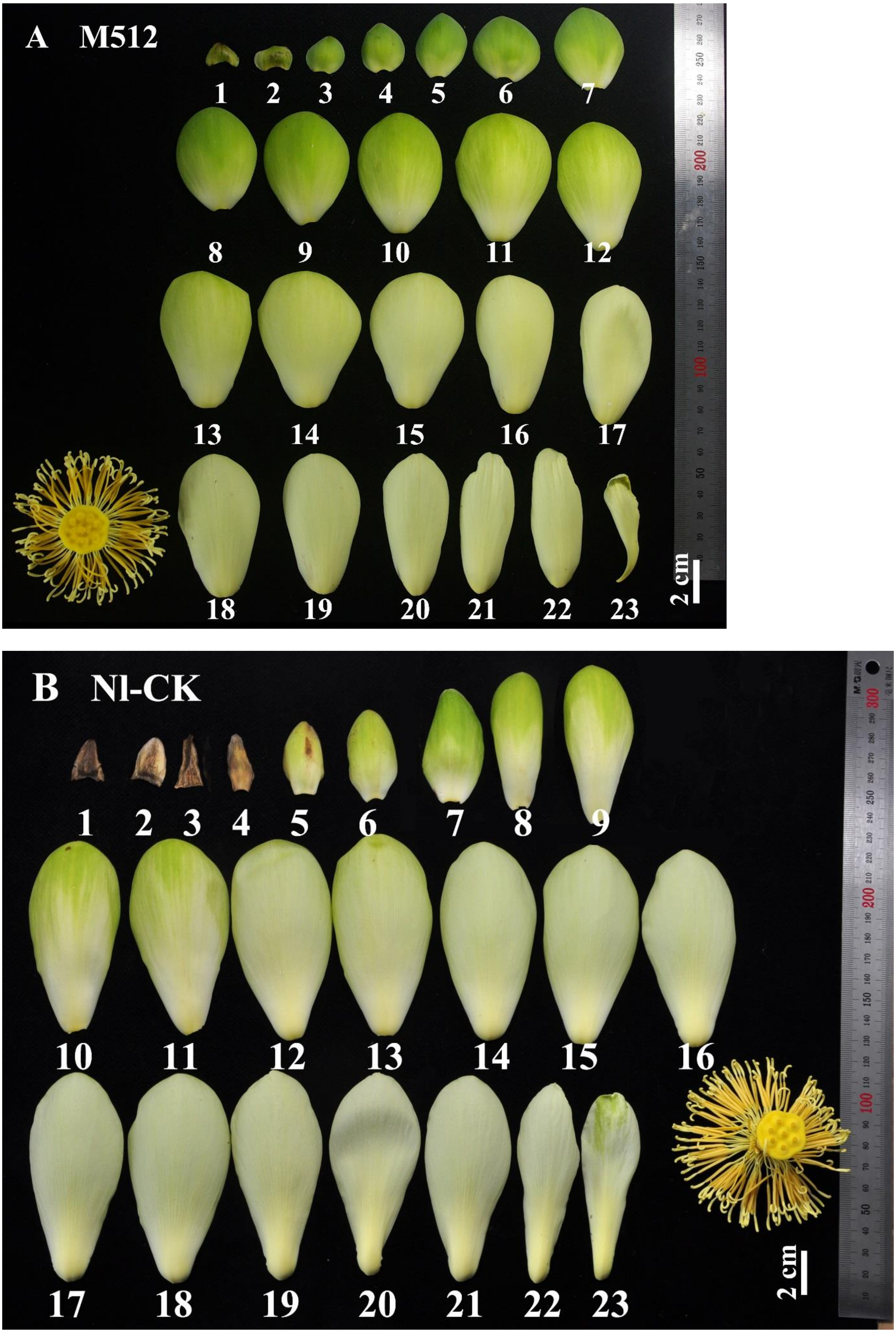

Supplementary Figure 1. Petal morphology of the broad variant M512 (**A**) and the control NL-CK (**B**) on the day of flowering (D15).

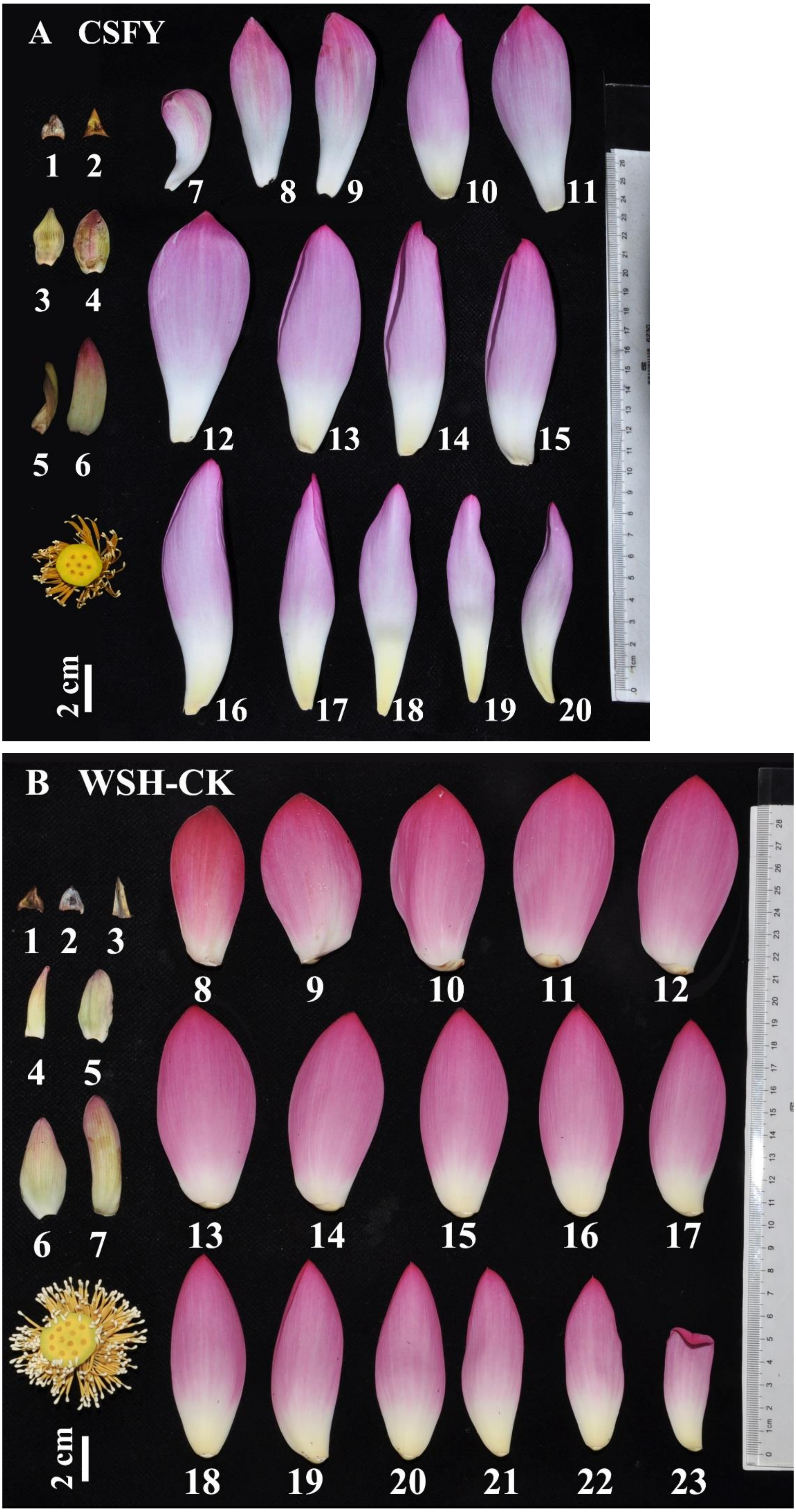

Supplementary Figure 2. Petal morphology of the narrow variant CSFY (**A**) and WSH-CK (**B**) on the day of flowering (D15).

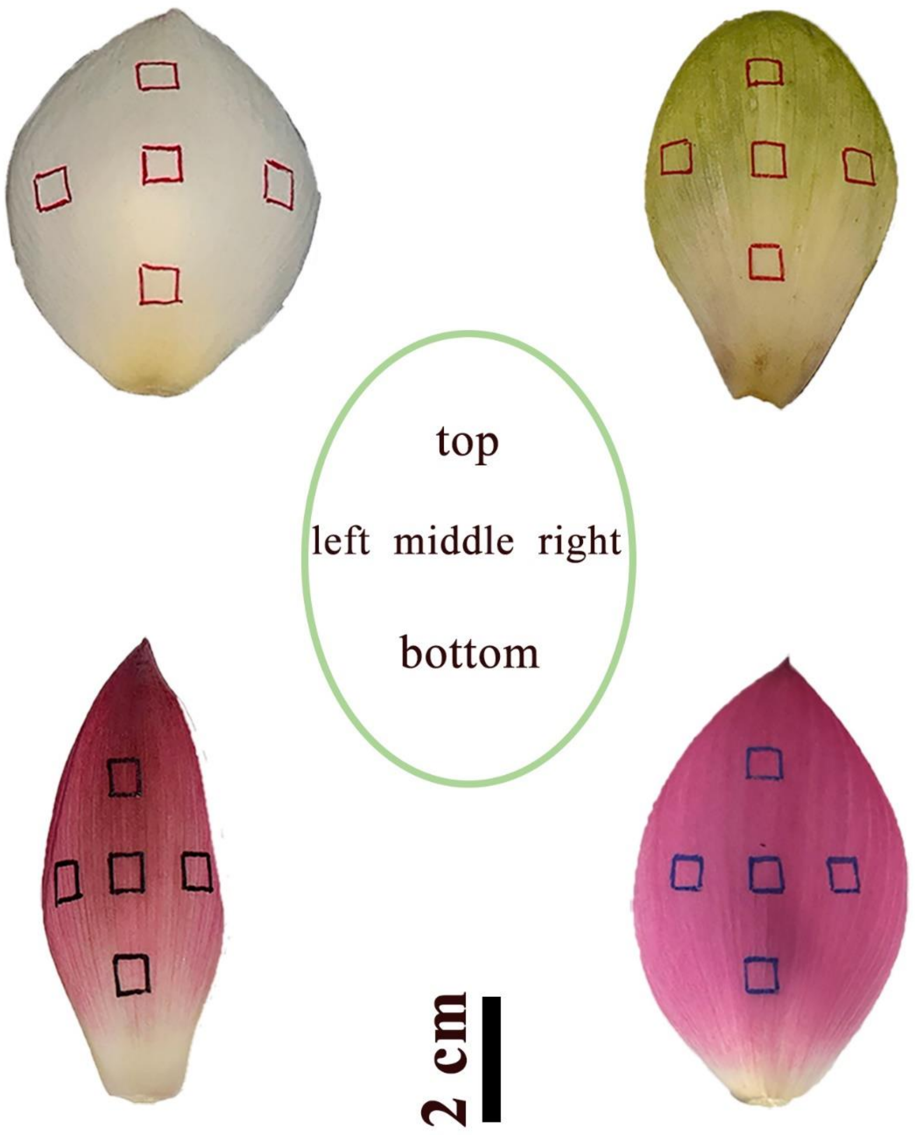

Supplementary Figure 3. Five sampling positions for microscopic observation of epidermal cell count (density) on lotus petals

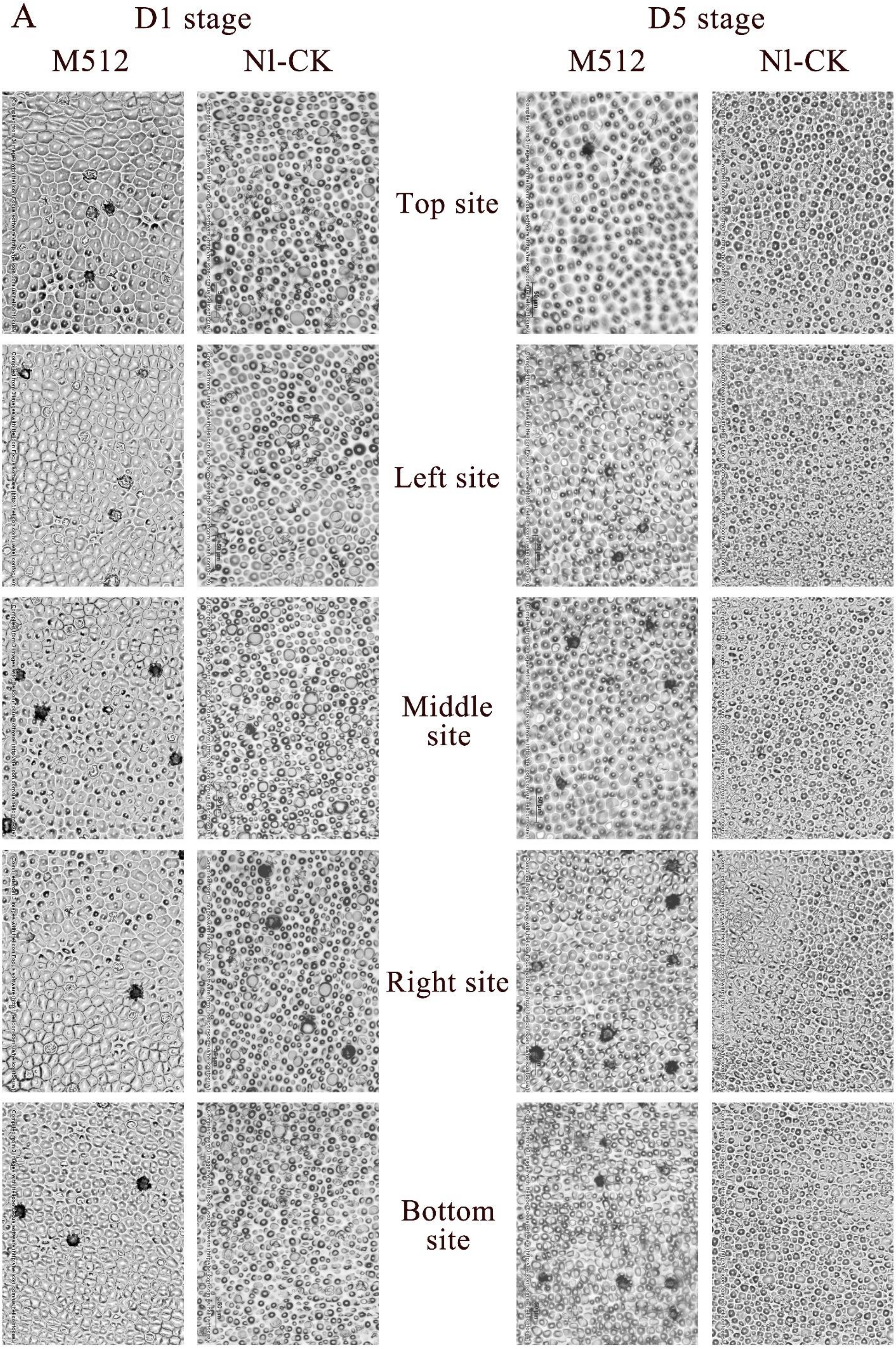
A
D1 stage
D5 stage
M512
Nl-CK
M512
Nl-CK
Top site
Left site
Middle site
Right site
Bottom site

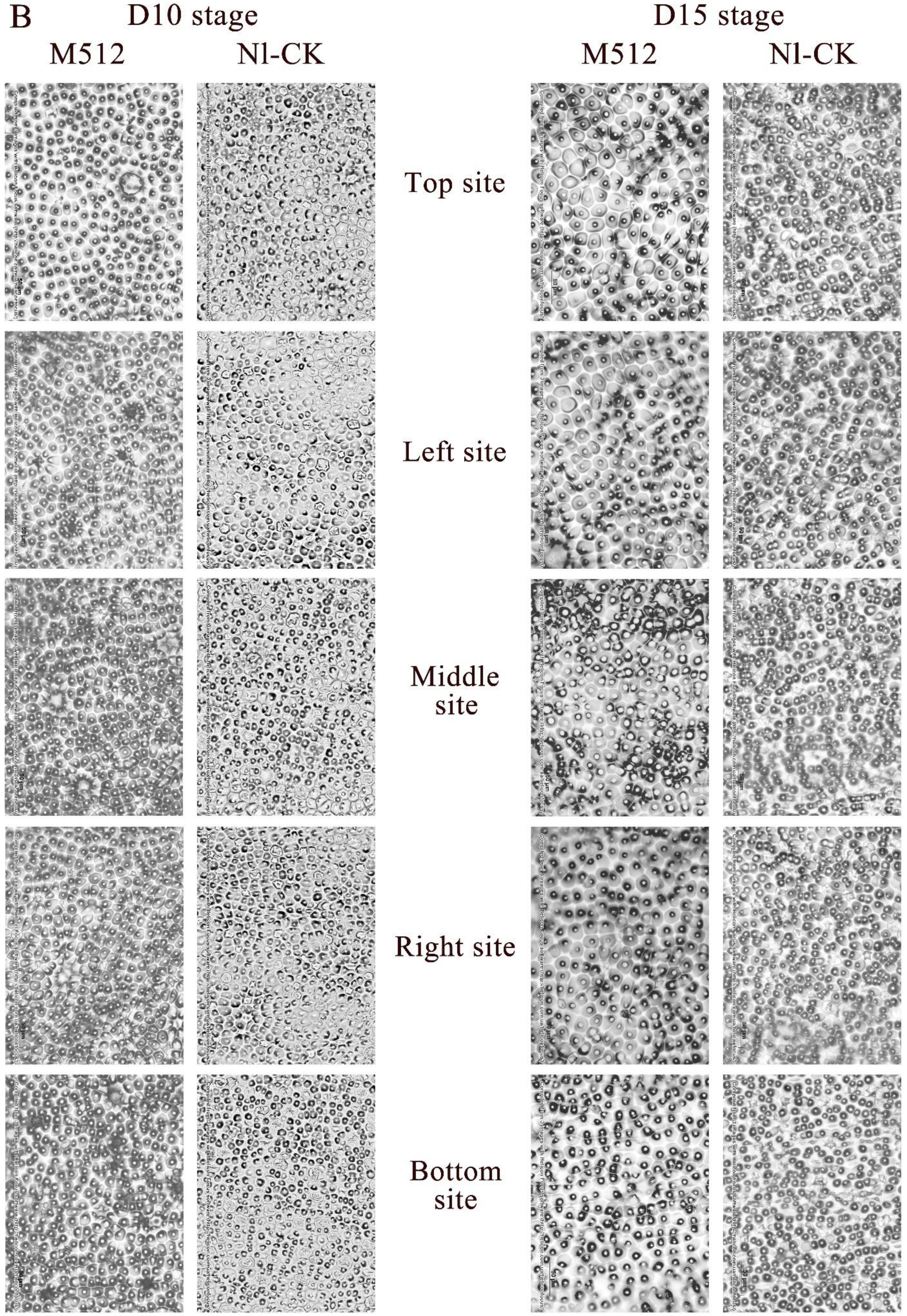

Supplementary Figure 4. Microscopic morphology of epidermal cells in M512 and NL-CK petals at D1 and D5 stages (**A**) and at D10 and D15 stages (**B**). (40x objective). The original images can be found in Supplementary Material 1.

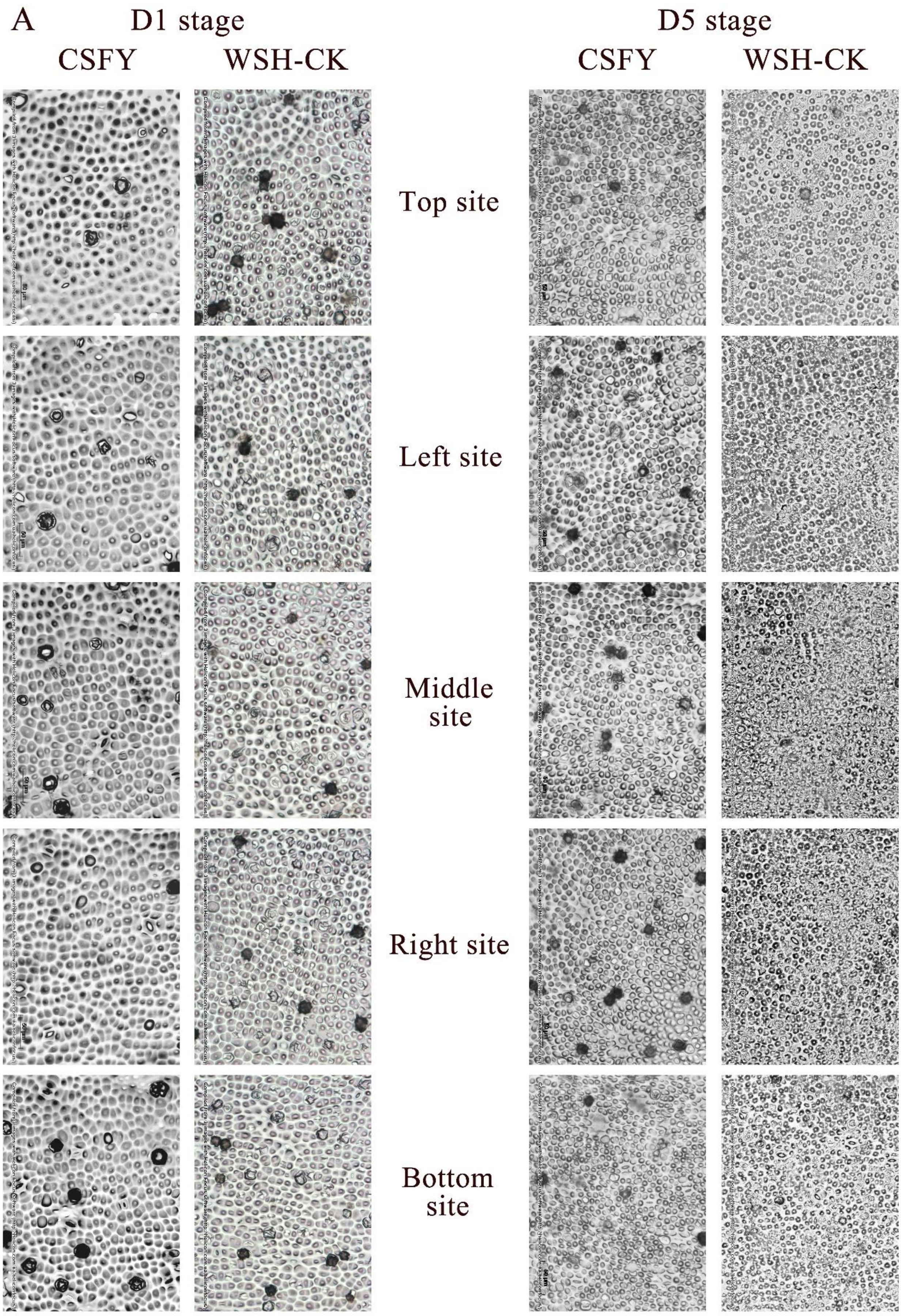
A
D1 stage
CSFY
WSH-CK
D5 stage
CSFY
WSH-CK
Top site
Left site
Middle site
Right site
Bottom site

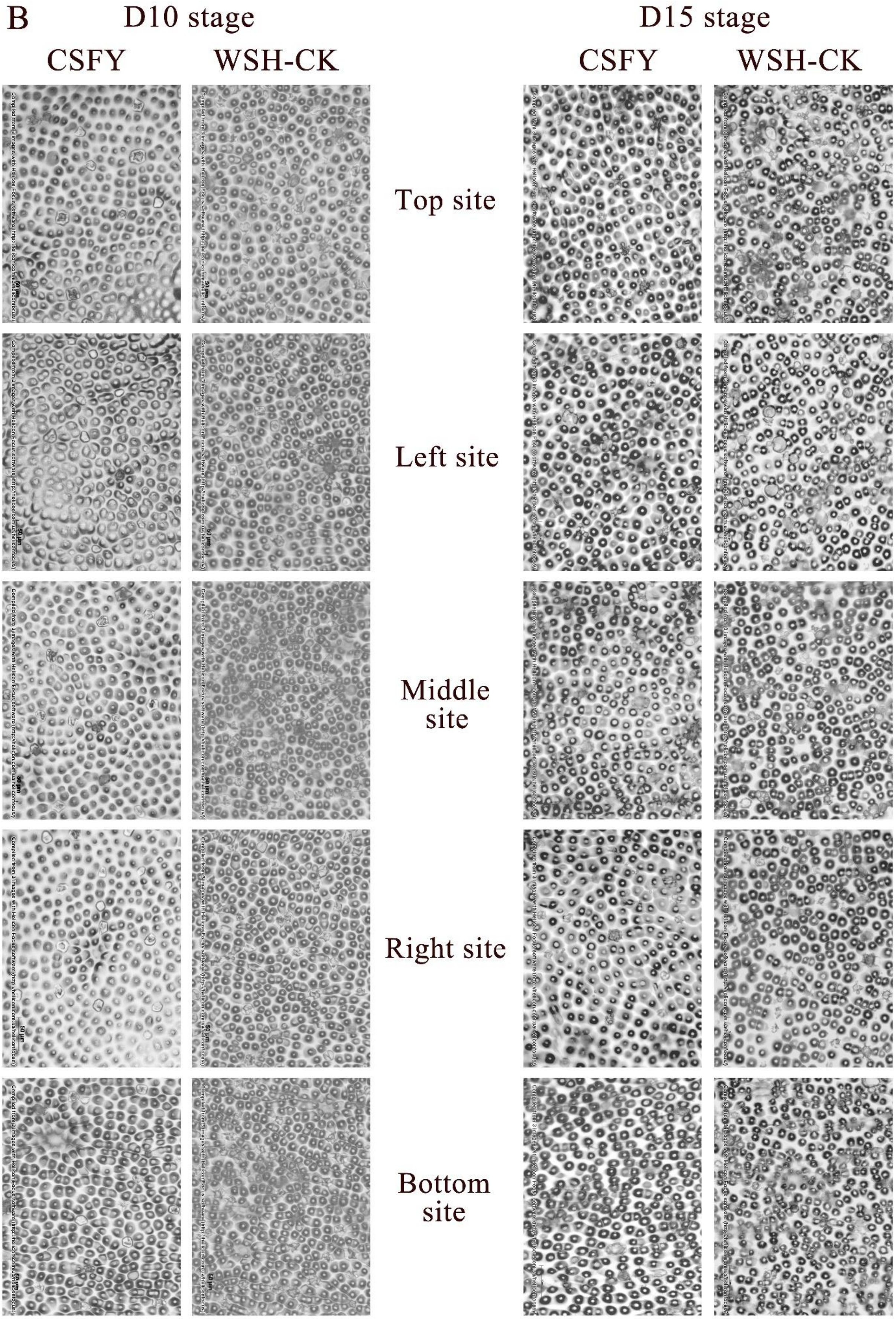

Supplementary Figure 5. Microscopic morphology of epidermal cells in CSFY and WSH-CK petals at D1 and D5 stages (**A**) and at D10 and D15 stages (**B**). (40x objective). The original images can be found in Supplementary Material 1.

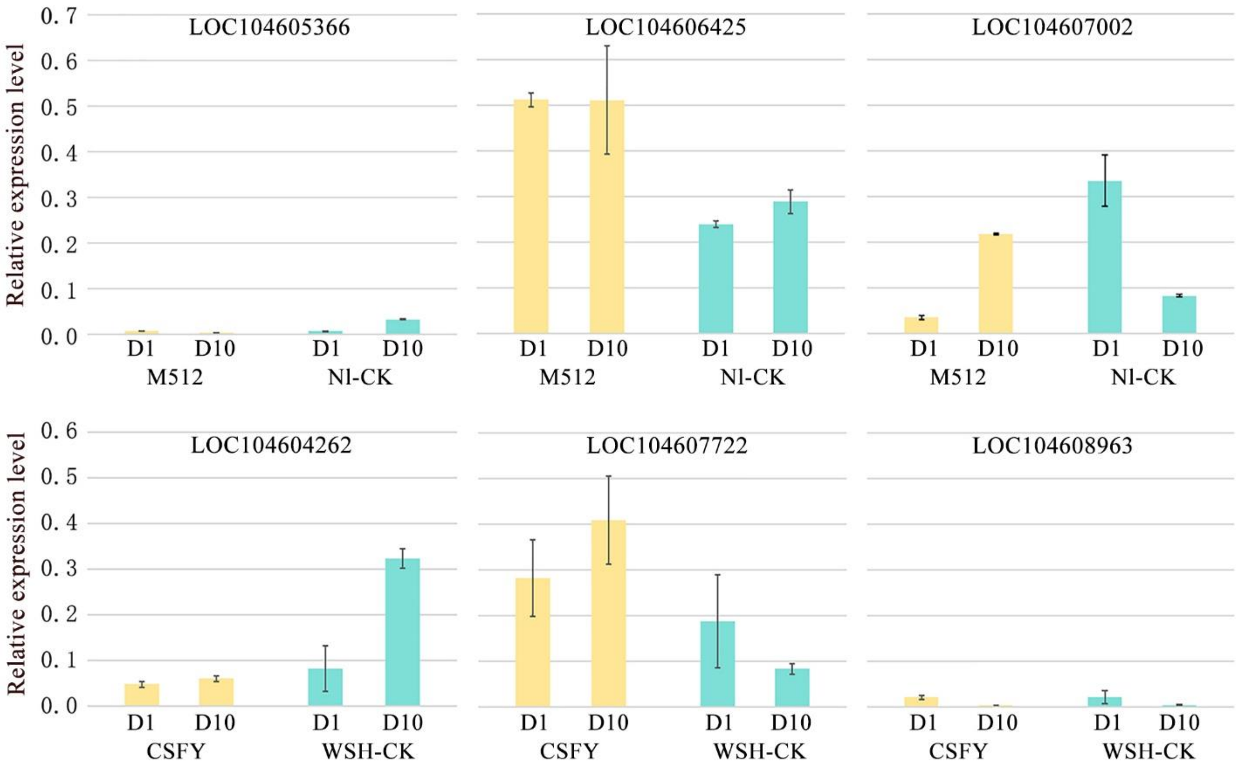

Supplementary Figure 6. RT-qPCR validation of the expression levels of six randomly selected differentially expressed genes (DEGs).
